# ATM mutations and E-cadherin expression define sensitivity to EGFR-targeted therapy in colorectal cancer

**DOI:** 10.18632/oncotarget.15211

**Published:** 2017-02-09

**Authors:** Anna-Lena Geißler, Miriam Geißler, Daniel Kottmann, Lisa Lutz, Christiane D. Fichter, Ralph Fritsch, Britta Weddeling, Frank Makowiec, Martin Werner, Silke Lassmann

**Affiliations:** ^1^ Institute of Surgical Pathology, University of Freiburg, Freiburg im Breisgau, Germany; ^2^ Faculty of Medicine, University of Freiburg, Freiburg im Breisgau, Germany; ^3^ Faculty of Biology, University of Freiburg, Freiburg im Breisgau, Germany; ^4^ German Cancer Consortium (DKTK) and German Cancer Research Center (DKFZ), Heidelberg, Germany; ^5^ Department of Internal Medicine, University of Freiburg, Freiburg im Breisgau, Germany; ^6^ Department of Surgery, University of Freiburg, Freiburg im Breisgau, Germany; ^7^ Comprehensive Cancer Center Freiburg, All Medical Center – University of Freiburg, Freiburg im Breisgau, Germany; ^8^ BIOSS Centre for Biological Signaling Studies, University of Freiburg, Freiburg im Breisgau, Germany

**Keywords:** anti-EGFR therapy, next generation sequencing, predictive markers, colorectal cancer (CRC), E-cadherin

## Abstract

EGFR-targeted therapy is a key treatment approach in patients with RAS wildtype metastatic colorectal cancers (CRC). Still, also RAS wildtype CRC may be resistant to EGFR-targeted therapy, with few predictive markers available for improved stratification of patients. Here, we investigated response of 7 CRC cell lines (Caco-2, DLD1, HCT116, HT29, LS174T, RKO, SW480) to Cetuximab and correlated this to NGS-based mutation profiles, EGFR promoter methylation and EGFR expression status as well as to E-cadherin expression. Moreover, tissue specimens of primary and/or recurrent tumors as well as liver and/or lung metastases of 25 CRC patients having received Cetuximab and/or Panitumumab were examined for the same molecular markers. *In vitro* and *in situ* analyses showed that EGFR promoter methylation and EGFR expression as well as the MSI and or CIMP-type status did not guide treatment responses. In fact, EGFR-targeted treatment responses were also observed in RAS exon 2 p.G13 mutated CRC cell lines or CRC cases and were further linked to PIK3CA exon 9 mutations. In contrast, non-response to EGFR-targeted treatment was associated with ATM mutations and low E-cadherin expression. Moreover, down-regulation of E-cadherin by siRNA in otherwise Cetuximab responding E-cadherin positive cells abrogated their response. Hence, we here identify ATM and E-cadherin expression as potential novel supportive predictive markers for EGFR-targeted therapy.

## INTRODUCTION

Colorectal cancer (CRC) is the third most common cancer and the third leading cause of cancer death in both men and women [1]. Although death rates are declining due to early detection programs, improved treatment strategies for metastatic CRC are still emerging. Surgical resection and/or chemotherapy based on capecitabine, irinotecan, oxaliplatin, fluorouracil, leucovorin and targeting drugs like bevacizumab are a current standard therapeutic option [2]. Moreover, monoclonal antibodies (mAbs) targeting the epidermal growth factor receptor (EGFR), namely Cetuximab and Panitumumab, have been approved for the metastatic situation. These therapeutic mAbs bind to the extracellular domain of EGFR and inhibit downstream signaling of the RAS/MAPK and PI3K/AKT pathways, which promote (cancer) cell proliferation, survival and growth. Resistance to EGFR-targeting mAbs exists in CRC patients, whose tumors harbor an EGFR downstream activating KRAS or NRAS mutation. Hence, RAS mutation testing is a routine diagnostic molecular pathology pre-requisite for clinical decision making on EGFR-targeted therapy, as laid out in clinical guidelines [3]. Still, 40-60% of CRC patients with RAS wild type tumors do not benefit from EGFR-targeted mAbs [4] and predictive markers for this subgroup of CRC patients are lacking. Since EGFR-targeted therapy is mostly in combination with chemotherapy (e.g. FOLFOX treatment regimen), not only EGFR-associated, but also other cellular signaling pathways and processes may confer resistance and define novel predictive markers.

Indeed, predictive markers further to RAS were first suggested to be found within the EGFR downstream signaling pathways, including alterations of - for example - BRAF, PIK3CA and PTEN [5, 6]. Of these, PIK3CA and associated kinases - as key regulators of the PI3K/AKT pathway - are highly attractive predictive and, in fact, also potentially actionable candidates: PIK3CA is a proto-oncogene encoding phosphatidylinositol 3-kinase (PI3K), the signal inducer of the PI3K-AKT pathway and is mutated in about 10-30% of CRC, mostly in sequence hotspots in exons 9 and 20 [7, 8]. It is predictive for inhibition by combined Dabrafenib/Trametinib treatment [9] and is itself targetable by novel agents.

Nevertheless, predictive markers for CRC patients scheduled for or undergoing EGFR-targeted (chemotherapy-combined) therapy may also reside within the cellular mechanisms of cell damage. As such, ataxia-telangiectasia mutated (ATM) is a serine/threonine protein kinase belonging to the PI3K family. It functions as a key mediator of DNA damage response and induces cell cycle arrest [10]. Furthermore, inhibitors against ATM are in preclinical development and a radiosensitizing effect was reported, particularly in cells defective in p53 [11]. Both, ATM and TP53 are frequently mutated in cancers of the colon and rectum [12], but their predictive value for RAS wildtype CRC patients treated by EGFR-targeted therapy in combination with chemotherapy still has to be elucidated.

Irrespective of these “downstream” mechanisms of treatment resistance and potential predictive markers, few studies have (re-)evaluated alterations within the actual target structure - EGFR - and/or its altered expression or functionality. Hence, in contrast to the prediction of treatment responses to HER2-targeted mAb therapy in breast cancer [13], EGFR protein expression analysis or its regulation at the DNA level are not in the focus of treatment prediction of response to EGFR-targeted therapy in CRC patients. Moreover, the role of crosstalk of EGFR with other cellular pathways and/or induced EGFR by-pass signaling via e.g. other membrane receptors [14] remains unclear.

With the increasing knowledge about epigenetic regulation of cancers, methylation of CpG sites within promoter regions may be an attractive monitor of gene repression and protein expression [15]. So far, few studies have addressed the role of EGFR methylation status as a predictive marker for EGFR-targeted treatment responses beyond the key predictive markers addressing gene mutations and yielded conflicting results: Scartozzi [16] described a better overall survival in patients without EGFR promoter methylation, whereas Chiadini [17] reported an improved response and overall survival in patients with ≥10% EGFR methylation.

Protein expression and/or activity of EGFR - thereby also response to EGFR-targeted therapies - may also be regulated by other means, for example E-cadherin: E-cadherin is a transmembrane protein, playing a role in cell adhesion and the maintenance of epithelial cell integrity, which is altered upon metastasis [18]. E-cadherin is known to be lost in epithelial cancers during epithelial to mesenchymal transition (EMT) and metastasis [19], such as in CRC [20] and non-small-cell lung cancers [21]. Interestingly, in an experimental model system, soluble E-cadherin was induced by MMP9 activity and - in its soluble form – was able to actually activate EGFR [22]. Thus, the observed “loss” of E-cadherin - as detected by immunohistochemistry in most studies so far - may in fact indicate the induction of a soluble E-cadherin form, which interacts with and activates EGFR, thereby possibly leading to treatment resistance. Indeed, correlation of E-cadherin protein expression in CRC [23, 24], NSCLC [25] and urothelial [26] cancer cells with response to EGFR-targeted therapy showed that E-cadherin “loss” (with possible induction of soluble E-cadherin) points towards EGFR-targeted treatment resistance.

In the present study, we therefore assessed broad mutation profiles, EGFR methylation and expression as well as E-cadherin expression as predictive markers in 7 CRC cell lines treated by EGFR-targeted mAb *in vitro* as well as in a cohort of 25 clinically RAS wildtype CRC patients having been treated by EGFR-targeted therapy. We identify mutations in DNA damage response associated genes and E-cadherin expression as potential supportive predictive markers for EGFR-targeted therapy of RAS wildtype CRC.

## RESULTS

### Sensitivity of CRC cell lines to Cetuximab

To establish *in vitro* correlates for EGFR-targeted therapy responses observed in CRC patients, we first measured the effect of Cetuximab on cell viability of seven colorectal cancer (CRC) cell lines. Of these, 3/7 cell lines are KRAS and NRAS wild type (Caco-2, HT29 and RKO) and 4/7 cell lines are KRAS mutated (DLD1, HCT116, LS174T and SW480). In addition, 3/7 cell lines are microsatellite stable (Caco-2, HT29, SW480) and 4/7 are microsatellite instable (DLD1, HCT116, LS174T, RKO) [27]. For further molecular classification, CpG island methylator phenotype (CIMP) status determination revealed CIMP positivity for 4/7 cell lines (DLD1, HCT116, HT29 and RKO) and CIMP negativity for 3/7 cell lines (Caco-2, LS174T and SW480).

**Figure 1:**
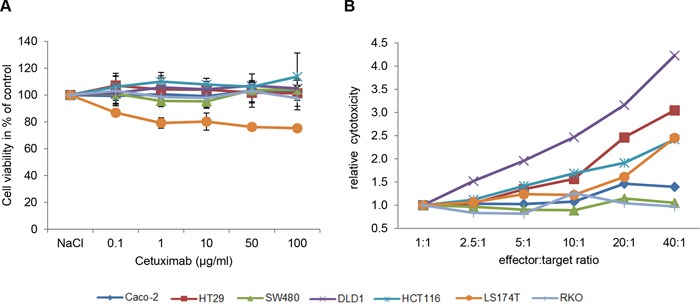
Cetuximab elicits antibody-dependent cellular cytotoxicity in CRC cell lines **A**. Three KRAS wild type (Caco-2, HT29, RKO) and four KRAS mutant cell lines (SW480, DLD1, HCT116, LS174T) were treated with increasing concentrations of Cetuximab (0.1, 1, 10, 50, 100 μg/ml) for 72h. Cell viability was measured using MTS assay. Cell viability is illustrated in % of control cells (0.9% NaCl). Values shown are the mean of n=3 independent experiments. **B**. Antibody dependent cellular cytotoxicity (ADCC) measured after co-cultivation of increasing numbers of effector cells (peripheral blood mononuclear cells, PBMCs) with target cells (CRC cell lines) for 4h in the presence of 10μg/ml Cetuximab.

As expected for mAb-based treatment *in vitro*, mere incubation of CRC cells with different concentrations of Cetuximab (0.1, 1, 10, 50 and 100 μg/ml) had almost no effect on cell viability in any of the investigated CRC cell lines. In this setting, the strongest response was a 20% loss of cell viability in exclusively LS174T cells (Figure 1A).

We therefore next investigated the response of the seven CRC cell lines to Cetuximab by antibody-dependent cellular cytotoxicity (ADCC) [28, 29]. Indeed, upon co-culture with increasing numbers of effector cells, three distinct patterns of response to Cetuximab were observed in the seven CRC cell lines (Figure 1B): Cetuximab was most effective in DLD1 cells, followed by intermediate responses in HT29, HCT116, LS174T and Caco-2 cells, whereas RKO and SW480 cells were non-responsive to Cetuximab treatment at all effector-target ratios. Thereby, the cell line responses to Cetuximab were not directly linked to RAS mutation status, with responding cells either being RAS mutated (DLD1, HCT116, LS147T) or not (Caco-2, HT29). Vice-versa, non-responding cells were either RAS mutated (SW480) or not (RKO).

Hence, the selected CRC cell lines are a model for EGFR-targeted treatment by mAbs *in vitro* and - as seen in CRC patients - their RAS mutation status does not appear to be the single predictive marker for treatment response to EGFR-targeted mAb therapy.

### Distinct mutation profiles occur in Cetuximab responding and non-responding CRC cell lines

Screening for 46 additional genes to KRAS and NRAS by targeted next generation sequencing next defined additional oncogenes and/or tumor suppressor genes related to the observed Cetuximab responses *in vitro*.

The number of mutated genes in the 7 cell lines ranged from 4 (Caco-2) to 10 (DLD1) (Supplementary Figure 1A). No significant mutation pattern correlating with the cell lines’ response or non-response to Cetuximab was detected. However, the resistant cell lines RKO and SW480 both harbored an ATM mutation and no mutations in those genes affected in the responding and intermediate responding CRC cell lines (HNF1A, SMAD4, SMO, ABL, CTNNB1, IDH1, NOTCH1, STK11) (Supplementary Figure 1B). In addition, PIK3CA exon 9 mutations (p.E545K, p.D549N) were detected exclusively in responding DLD1 cells, whereas a PIK3CA exon 20 mutation (p.H1047R) was present in intermediate responding HCT116 and LS174T as well as in non-responding RKO cell (Table 1).

**Table 1:** KRAS, BRAF PIK3CA and ATM mutations of 7 CRC cell lines

Cell line	Cetuximab response	KRAS	BRAF	PIK3CA	ATM
**DLD1**	+++	p.Gly13Asp		p.Glu545Lys p.Asp549Asn	
**HCT116**	++	p.Gly13Asp		p. His1047Arg	
**LS174T**	++	p.Gly12Asp		p.His1047Arg	
**SW480**	-	p.Gly12Val			p.Arg2461Pro
**Caco-2**	++				
**HT29**	++		p.Val600Glu		
**RKO**	-		p.Val600Glu	p.His1047Arg	p.Pro872Ser

Finally, all cell lines were screened for the recently described EGFR exon 12 resistance mutation p.S492R, described as occurring during EGFR-treatment [30] by dideoxy sequencing. However, none of the cell lines was positive for this mutation at base-line (Supplementary Figure 1C).

Thus, broader mutation profiles or the number of mutations does not alone define response of CRC cells to Cetuximab *in vitro*.

### Regulation of EGFR expression by EGFR promoter methylation

**Figure 2:**
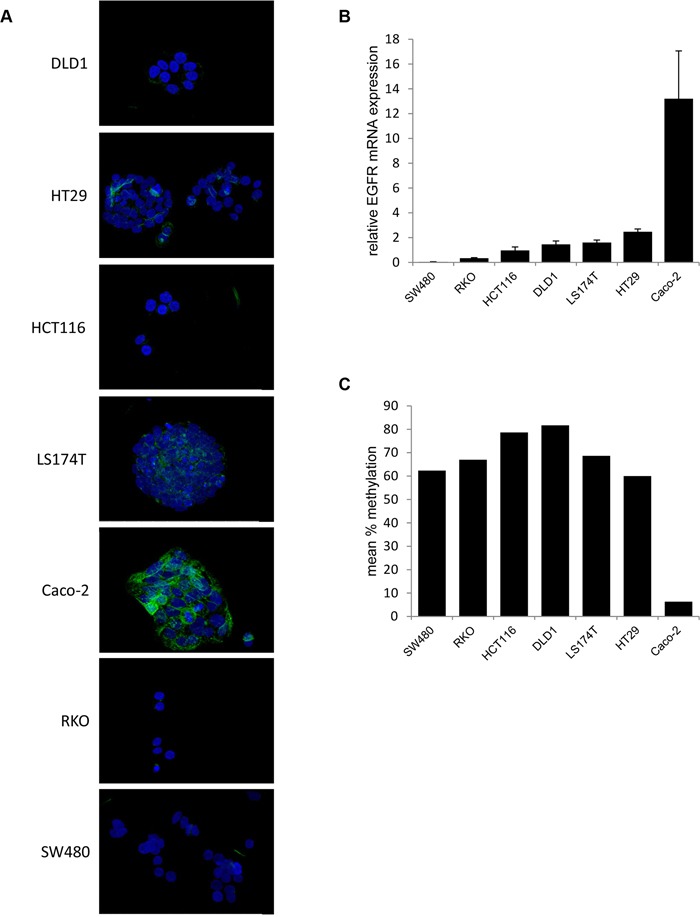
EGFR expression is inversely correlated with EGFR promoter methylation in CRC cell lines **A**. Colorectal cancer cell lines (SW480, RKO, HCT116, DLD1, LS174T, HT29 and Caco-2) were stained for EGFR (green) and DAPI for visualization of the nucleus (blue). The representative stainings show a 40x magnification. **B**. Relative EGFR mRNA expression as determined by q-RT-PCR (mean ± standard deviation of three independent experiments; relative to a universal reference RNA). **C**. Mean % methylation of three CpG sites within the promoter of EGFR.

To correlate *in vitro* Cetuximab treatment responses to potential alterations of the target structure, i.e. EGFR itself, EGFR mRNA and protein expression as well as EGFR promoter methylation were assessed in all seven CRC cell lines (Figure 2).

Immunofluorescence revealed strong membranous EGFR protein expression only in Caco-2 cells (Figure 2A). Marginal, mainly cytoplasmic EGFR protein expression was observed in HT29, LS174T and DLD1 cells, whereas the HCT116, RKO and SW480 cells were EGFR negative. These EGFR protein expression patterns correlated to EGFR mRNA expression, which was highest in Caco-2 (13.21±3.85) cells, followed by HT29 (2.47±0.23), LS174T (1.60±0.20), DLD1 (1.45±0.28), HCT116 (0.97±0.28), RKO (0.34±0.04) and SW480 (0.04±0.02) cells (Figure 2B).

Finally, epigenetic regulation of EGFR expression [31] was examined by EGFR promoter methylation analysis via pyrosequencing. EGFR promoter methylation was lowest in the strong EGFR expressing Caco-2 cells (6.3%) and higher (range 60%-81%) in all other CRC cell lines (Figure 2C).

Hence, in addition to RAS status also EGFR expression, closely regulated by DNA promoter methylation in Caco-2 cells, does not directly guide the responses of CRC cell lines to Cetuximab.

### E-cadherin protein expression differs in Cetuximab responding and non-responding CRC cell lines

Based on the hypothesis that E-cadherin expression may influence EGFR-targeted treatment responses [24–26], we next examined E-cadherin protein expression in all seven CRC cell lines.

**Figure 3:**
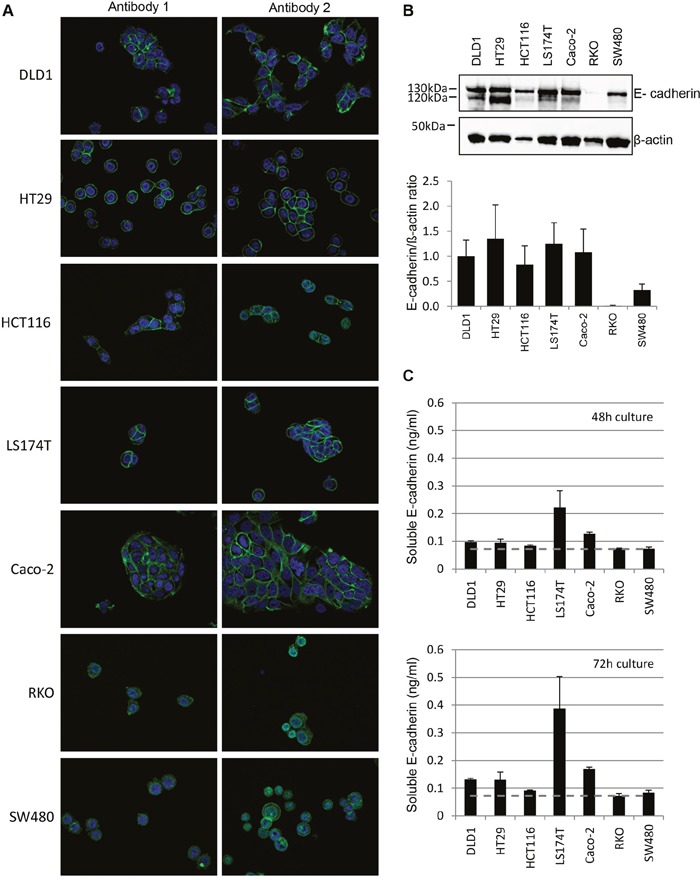
Distinct E-cadherin expression in Cetuximab responding, intermediate-responding and non-responding CRC cell lines **A**. CRC cells were stained for E-cadherin (green) and DAPI for visualization of the nucleus (blue). Stainings were performed by two antibodies, with antibody 1= clone NCH-38 (DakoCytomation/Agilent), detecting the 120kDa mature and a 82kDa soluble E-cadherin, and antibody 2= clone 24E10 (Cell Signaling Technology) raised against P790 of E-cadherin. The representative stainings show a 40x magnification. **B**. Top:Representative immunoblot for E-cadherin and β-actin. Note that western blot analysis detects a double band for E-cadherin in DLD1, HT29, LS174T and Caco-2 cells, which correlates to mature (120kDa) and immature (130kDa) E-cadherin. Bottom: Quantification of E-cadherin protein levels. Protein expression normalized to β-actin. **C**. Quantification of soluble E-cadherin measured in three independent experiments at 48h (top) and 72h (bottom) by ELISA. Note that soluble E-cadherin levels were increased upon 72h of cultivation and are highest in LS174T followed by Caco-2, DLD1 and HT29 cells.

As seen by immunofluorescence staining using two E-cadherin antibodies (Figure 3A), strong membranous and in part cytoplasmic E-Cadherin was detectable in DLD1 cells. HT29 and LS174T cells also showed marked fully circular membranous E-cadherin expression, whilst in Caco-2 and HCT116 E-cadherin expression was in part non-membranous and more cytoplasmic in cells without other cell contacts. In RKO and SW480 cells, weak E-cadherin expression was seen. In the latter two cell lines with weak E-cadherin expression as detected by the first antibody (clone NCH-38, DakoCytomation/Agilent, recognizes the 120 kD mature form and a 82kD (soluble) fragment of E-cadherin), the second antibody (clone 24E10, Cell Signaling Technology, raised against P780 of E-cadherin and stains cytoplasmic E-cadherin) revealed a stronger E-cadherin expression, but predominantly in the cytoplasm. The levels of E-cadherin protein expression were confirmed by western blot analyses using the second antibody, showing lower E-cadherin protein levels in RKO and SW480 (Figure 3B). Moreover, whilst DLD1, HCT116, LS174T and Caco-2 cells expressed both the mature (120kDa) and immature (130kDa) E-cadherin protein, this was not the case for HCT116, RKO and SW480 cells.

To investigate soluble levels of E-cadherin, cell culture supernatants of the 7 cell lines were additionally examined by ELISA at 48 and 72 hours (Figure 3C). Thereby, low levels of soluble E-cadherin were detected in RKO and SW480 cells, whilst moderate soluble E-cadherin levels with a slight increase at 72 hours of cultivation were seen in ascending order in HCT116, DLD1, HT29, Caco-2 and LS174T cells.

**Figure 4:**
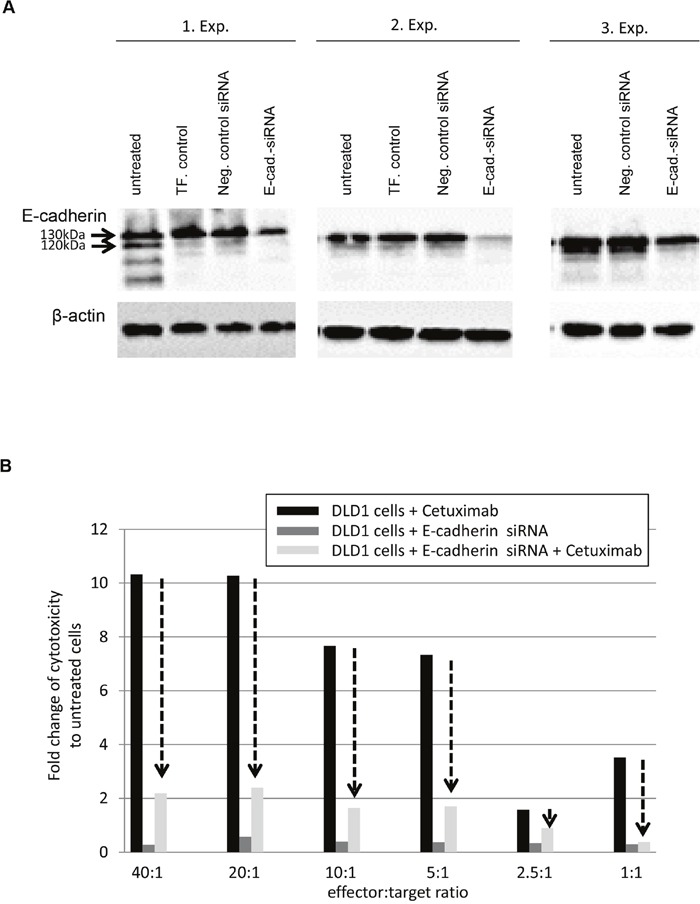
Down-regulation of E-cadherin abrogates response to Cetuximab in DLD1 cells **A**. Western blots show three independent experiments of E-cadherin-specific siRNA treatment with associated controls (untreated; TF=transfection control; Neg. Control siRNA= unspecific siRNA). Note the down-regulation of E-cadherin in its immature form (130kDa) and complete loss of its mature form (120kDa). **B**. Antibody dependent cellular cytotoxicity (ADCC) measured after co-cultivation of increasing numbers of effector cells (peripheral blood mononuclear cells, PBMCs) with control-siRNA or E-cadherin-siRNA trated DLD1 cells for 4h in the absence or presence of 10μg/ml Cetuximab. Data is presented as fold change of cell cytotoxicity relative to untreated (no Cetuximab, no siRNA) DLD1 cells (each set to 1, bars not shown). Note the loss of Cetuximab responses of DLD1 cells upon down-regulation of E-cadherin by siRNA (arrows).

To further validate that E-cadherin levels have an impact on the response of CRC cells to Cetuximab, DLD1 exhibiting strong E-cadherin protein expression were subjected to control- and E-cadherin-specific siRNA treatment followed by analysis of antibody-dependent cellular cytotoxicity. As shown in Figure 4A, E-cadherin protein levels were down-regulated post 72h of E-cadherin-specific siRNA treatment, particularly regarding the loss of the 120kDa mature E-cadherin. Moreover, in DLD1 cells treated by E-cadherin-specific siRNA and Cetuximab, the cell cytotoxicity via ADCC was impaired by 2- to 5-fold as compared to DLD1 cells treated by Cetuximab alone (Figure 4B). DLD1 cells treated with E-cadherin-siRNA alone did not show any significant cell cytotoxicity.

These data point towards a potential important role of E-cadherin in response to Cetuximab in CRC cell lines, since strong or weak membranous expression is associated with responding or non-responding CRC cell lines and since down-regulation of E-cadherin in responding DLD1 cells abrogates response to Cetuximab.

### The cohort of colorectal cancer patients receiving EGFR-targeted therapy

To translate the *in vitro* findings into a clinico-pathological situation, a cohort of 25 CRC patients was next investigated (Table 2). This included tissue specimen based analyses of primary tumors in 24/25 (96%) and a recurrent tumor in 1/25 (4%) of CRC cases as well as further case-matched analysis of recurrent tumors (n=2, derived from 2/25 CRC cases) and distant liver or lung metastases (n=20, derived from 12/25 CRC cases). All CRC patients had received EGFR-targeted therapy (n=13 Cetuximab; n=7 Panitumumab; n=5 Cetuximab and Panitumumab). This was combined with chemotherapy in 22/25 (88%) of cases. In three cases (#5, 15, 19) EGFR-targeted therapy was given prior to resection of the primary tumor (#5), recurrent tumor (#19) or liver metastasis (#15). According to the guidelines for initially only KRAS exon 2 and in recent years entire RAS [32] testing, all cases were wildtype for all 25 CRC cases at the time of clinical presentation. Diagnostic reports (external: 2/25; internal: 23/25) of KRAS exon 2 or RAS status were based on dideoxy sequencing in 24/25 CRC cases and on next generation sequencing in 1/25 CRC cases. For further molecular classification, microsatellite instability (MSI) and CpG island methylator phenotype (CIMP, Supplementary Figure 2) status was defined for the 25 CRC cases. This revealed 1/25 (4%) MSI-positive and 2/25 (8%) CIMP-positive CRC cases, with 1/25 (4%) being MSI- and CIMP-positive. All clinico-pathological (including clinical response criteria such as complete or partial response, stable or progressive disease, survival) and molecular (MSI, CIMP) data are given in Table 2.

**Table 2:** Detailed patients' characteristics

ID	sex	age	TU/M	tumor site	pT	pN	G	CIMP	MSI MSS	EGFR-targeted Tx	concurrent Tx	Response rate	OS (months)
**1**	f	63	PT M(HEP) M(HEP)	rectum	3	0	2	negative	MSS	Cetuximab Panitumumab	Bevacizumab Capecitabine Xelox	SD/PD	26
**2**	m	69	PT ReT	rectum	2	0	2	negative	MSS	Panitumumab		PD	1
**3**	m	58	PT M(HEP)	rectum	3	2	2	negative	MSS	Cetuximab Panitumumab	Bevacizumab FOLFOX FOLFIRI	PR/CR/PD	89
**4**	m	70	PT	colon	3	0	2	negative	MSS	Cetuximab	Irinotecan	PR/PD	10
**5**	f	60	PT	colon	4	0	3	negative	MSS	Cetuximab	FOLFOXIRI	PR/CR	
**6**	m	69	PT	colon	3	1	2	negative	MSS	Cetuximab Panitumumab	Irinotecan	PR/PD	13
**7**	f	66	PT	rectum	3	0		negative	MSS	Cetuximab	FOLFIRI	SD	
**8**	f	61	PT M(HEP)	colon	4	1	2	negative	MSS	Cetuximab Panitumumab	Irinotecan Capiri	SD/PD	38
**9**	m	37	PT M(PUL) M(PUL) M(HEP) M(PUL)	rectum	3	2	2	negative	MSS	Cetuximab	Irinotecan	SD/PD	15
**10**	m	75	PT	rectum	3	0	2	negative	MSS	Cetuximab	Irinotecan	PD	8
**11**	f	77	PT	colon	4	2	2	negative	MSS	Panitumumab	FOLFIRI	PD	0.5
**12**	m	71	PT M(HEP)	colon	3	2	2	negative	MSS	Panitumumab	FOLFOX	PR	
**13**	m	64	PT M(HEP)	colon	3	1	2	negative	MSS	Cetuximab	Bevacizumab Irinotecan FOLFOX FOLFIRI	SD/PD	10
**14**	f	65	PT M(HEP)	colon	4	2	2	negative	MSS	Cetuximab	Irinotecan	PD	6
**15**	f	63	PT ReT M(HEP)	rectum/sigma	2	1	2	negative	MSS	Cetuximab	FOLFIRI	CR/PR	28
**16**	f	70	PT M(HEP) M(HEP)	colon	4	1	3	negative	MSS	Panitumumab		PD	7
**17**	m	46	PT M(HEP)	colon	3	2	2	na	MSS	Cetuximab	Irinotecan	PR/PD	11
**18**	m	74	PT	colon	3	2	3	positive (4/5)	MSI	Cetuximab	FOLFIRI	PD	5
**19**	m	73	ReT	colon	4	0	2	positive (3/5)	MSS	Panitumumab			
**20**	m	61	PT M(HEP) M(HEP)	rectum	3	1	2	negative	MSS	Cetuximab	FOLFIRI		
**21**	f	59	PT	colon	4	2	3	negative	MSS	Cetuximab	Irinotecan	PD	3
**22**	m	63	PT	colon	4	2	2	negative	MSS	Panitumumab	FOLFOX	PR/PD	
**23**	f	68	PT	rectum	3	2	2	negative	MSS	Cetuximab	Irinotecan	PD	20
**24**	m	41	PT	colon	3	2	2	negative	MSS	Panitumumab	FOLFOX		
**25**	m	59	PT M(HEP) M(PUL) M(PUL)	rectum	3	2	2	negative	MSS	Cetuximab Panitumumab	Bevacizumab	PR	

The table summarizes the major clinico-pathological parameters and molecular (MSI, CIMP) data of the investigated 25 cases. Capiri=Capecitabine, Irinotecan; Xelox=Capecitabine, Oxaliplatin; FOLFOX=Leucovorin, 5-FU, Oxaliplatin; FOLFIRI=Leucovorin, 5-FU, Irinotecan; FOLFOXIRI=Leucovorin, 5-FU, Oxaliplatin, Irinotecan. Clinical response data is given as SD=stable disease (responders), PD=progressive disease (non-responder), CR=complete response, PR=partial response as well as overall survival (length of time from start anti-EGFR treatment to death of patient).

### RAS mutation profiling by targeted next generation sequencing

To obtain a comprehensive and full picture of the RAS status of CRC cases treated by EGFR-targeted therapy at times when only KRAS exon 2 testing was required, we performed targeted next generation sequencing (tNGS), including all 24 primary tumors, 3 recurrent tumors and 20 distant liver or lung metastases.

The tNGS data underlines the necessity for comprehensive RAS testing in CRCs. In total, 32 KRAS, 11 NRAS and 5 HRAS sequence variants were detected in the 47 tissue specimens of the CRC cohort. Thereby, 8/25 CRC cases presented with RAS “hotspot” mutations, which were maintained for case-matched tissue specimens upon disease progression. These RAS mutations were either in KRAS exon 2 previously undetected by dideoxy sequencing (case #2), in NRAS exon 2 (case #1), in KRAS/NRAS exon 3 (cases #7, #14, #16, #21, #23), in HRAS exon 2 (case #22), or were other KRAS/NRAS sequence variants documented in COSMIC without known therapeutic relevance so far (case #2) (Table 3).

**Table 3:** RAS mutations detected by next generation sequencing

Case ID		Tissue specimen	Gene	Exon	Mutation	% allele frequency
**1**	2005	PT	NRAS	2	p.Gly13Arg	55.63
		M(HEP)	NRAS	2	p.Gly13Arg	46.54
		M(HEP)	NRAS	2	p.Gly13Arg	84.54
**2**	2012	PT	KRAS	2	p.Gly12Asp	61.39
			NRAS	2	p.Gly10Glu	10.3
	2013	ReT	KRAS	2	p.Gly12Asp	24.29
			KRAS	2	p.Ala18Thr	16.57
			KRAS	3	p.Glu63Lys	30.48
**7**	2012	PT	KRAS	3	p.Gln61Arg	39.72
**14**	2012	PT	KRAS	3	p.Gln61His	97.95
	2012	M(HEP)	KRAS	3	p.Gln61His	34.49
**16**	2010	PT	KRAS	3	p.Thr58Ile	36.69
		M(HEP)	KRAS	3	p.Thr58Ile	65.31
		M(HEP)	KRAS	3	p.Thr58Ile	19.9
**21**	2012	PT	KRAS	3	p.Gln61Lys	59.72
**22**	2014	PT	HRAS	2	p.Gly12Asp	14.8
**23**	2008	PT	KRAS	3	p.Gln61Leu	23.23

Hence, broader RAS testing and more sensitive technologies, such as tNGS, improve detection of possible K/N/HRAS resistance mutations for EGFR-targeted therapy.

### Broad mutation profiling reveals a high frequency of mutations in receptor tyrosine kinases and genes of the RAS/MAPK and PI3K/AKT pathways

In addition to RAS mutations, tNGS sequencing revealed novel insights into the mutation profile of the 47 tissue specimens from CRC patients treated by EGFR-targeted therapy.

**Figure 5:**
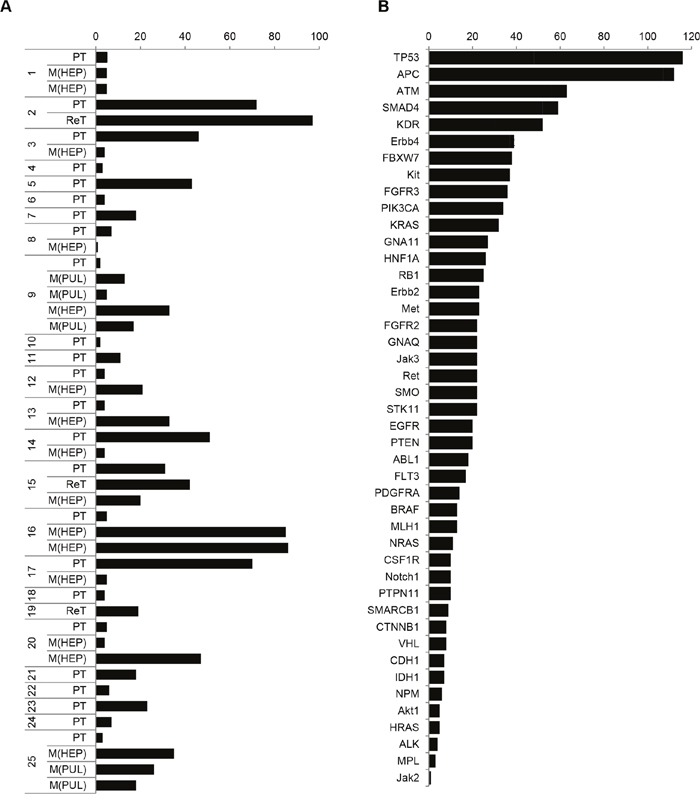
Case- and gene-specific frequency of detected variants is divergent within the CRC cohort **A**. Case-specific listing of the number of sequence variants. Missense, stop gained and frameshift mutations are included. **B**. Case-mixed listing of the mutated genes according to mutation frequency.

First, the mutational load showed inter- and intra-patient variability (Figure 5A): The number of mutations ranged from n=2 to n=97 mutations in 24 primary tumors and 3 recurrent tumors at time of initial clinical presentation, and from n=1 to n=86 mutations in 20 liver or lung metastases. In case-matched metastases, the mutational load was either maintained (1/12; 8.3% cases), increased (6/12; 50% cases) or decreased (5/12; 41.7% cases).

**Figure 6:**
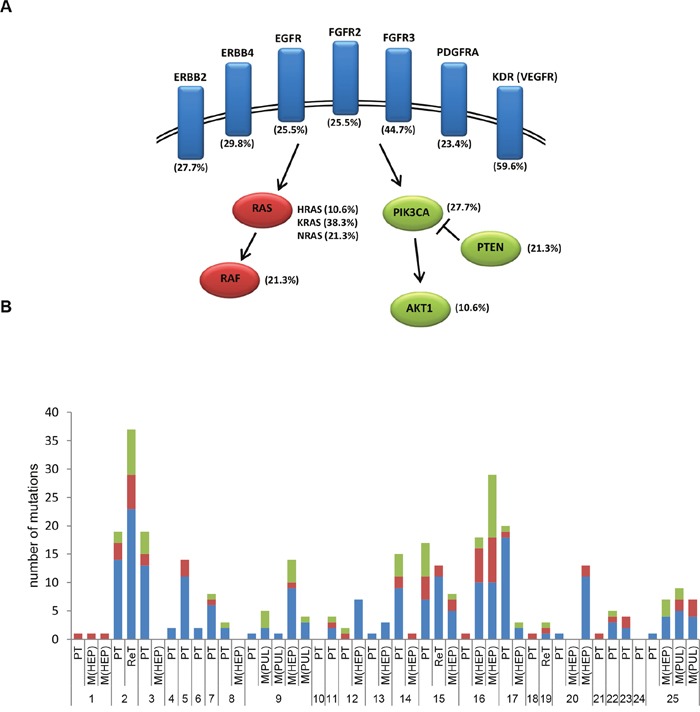
Mutations in receptor tyrosine kinases, RAS/MAPK and PI3K/AKT pathways in EGFR-treated CRC cases **A**. Frequency of detected mutations in receptor tyrosine kinases (highlighted in blue) and associated genes of the RAS/MAPK (highlighted in red) and PI3K/AKT (highlighted in green) pathways. **B**. Case-specific frequency of mutations in receptor tyrosine kinase (blue), RAS/MAPK (red) and PIK3/AKT (green) pathways.

Second, the most frequently mutated genes in all 47 analyzed tissue specimens were TP53 and APC, followed by mutations in ATM, SMAD4, KDR, ErBB4, FBXW7 and others, including KRAS, NRAS and HRAS (Figure 5B). Focusing on genes coding for receptor tyrosine kinases (including EGFR, ERBB2, ERBB4, FGFR2, FGFR3, PDGFRA, KDR/”VEGFR”) and associated genes of the RAS/MAPK and PI3K/AKT pathways the evaluation revealed mutations in EGFR in 25.5%, KRAS in 38.3%, BRAF in 21.3%, PI3KCA in 27.7%, AKT in 10.6% and PTEN in 21.3.% of cases (Figure 6A). Of these, all detected mutations in EGFR affect tyrosine kinase domains or ligand binding domains and were found widely distributed over EGFR exons 3,7,15,18,19,21 (Supplementary Table 1A). Since the recently described EGFR exon 12 p.G465 and p.S492 Cetuximab resistance mutations, described as occurring during EGFR-treatment [33], were not covered by tNGS, these were further determined by dideoxy sequencing. Neither EGFR exon 12 p.G465 nor p.S492 were mutated in the 25 CRC cases. However, a known single-nucleotide polymorphism was detected in EGFR intron 12/13 (dbSNP: rs1558544) in 7/25 CRC cases.

Finally, mutation profiles in EGFR signaling pathways were analyzed in a case-specific manner (Figure 6B; Supplementary Table 2): Within primary/recurrent tumors, exclusive receptor tyrosine kinase or RAS/MAPK mutations were observed in 6/25 (24%) or 4/25 (16%) of cases, respectively. In contrast, exclusive PI3K/AKT mutations were not detectable in primary/recurrent tumors, whereby mostly PIK3CA exon 9 and 20 mutations (Supplementary Figure 3, Supplementary Table 3) were seen in combination with other alterations. In 9/25 (36%) primary/recurrent tumors, mutations of receptor tyrosine kinases, RAS/MAPK and PI3K/AKT were present. In this setting, the receptor tyrosine kinases, RAS/MAPK and PI3K/AKT mutation profiles detected in the primary/recurrent tumors diverged upon metastasis in 9/12 (75%) of cases. Case-matched liver or lung metastases carried only receptor tyrosine kinase or RAS/MAPK mutations in 4/12 (16%) of cases and presented with mutations in receptor tyrosine kinases, RAS/MAPK and PIK3CA/AKT in 4/12 (16%) of cases.

These data show that complex mutation profiles may influence the response to EGFR-targeted therapy in CRC and possibly also the associated combination therapies.

### EGFR expression and EGFR promoter methylation in CRC

**Figure 7:**
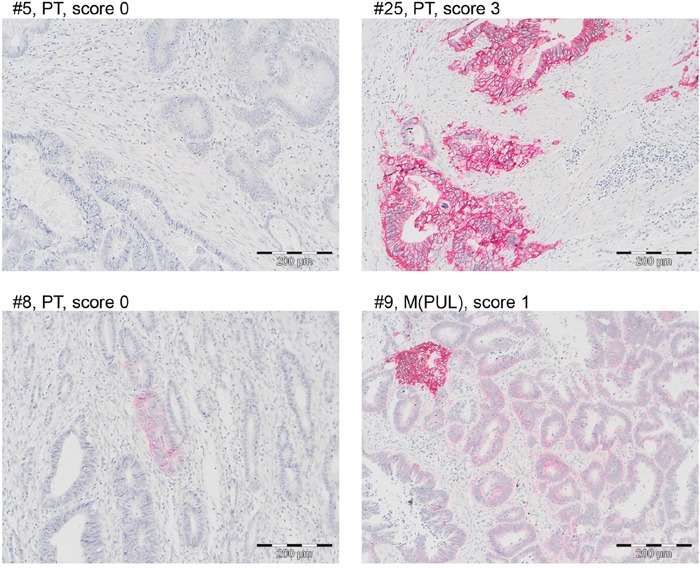
EGFR protein is rarely expressed in CRC tissue specimens A Representative EGFR stainings of four CRC cases with scores 0 (#5, #8), 1 (#9) and 3 (#25). Scoring was performed according to Fichter et al. [63]. Bar = 200 μm. Note that CRC cases #8 and #9 showed intratumoral and intraglandular heterogeneity.

To test whether *in vitro* observed EGFR expression and its regulation by DNA methylation is also seen *in situ*, EGFR promoter methylation, mRNA and protein expression were analyzed in 24 primary tumors, 3 recurrent tumors and 17 distant liver or lung metastases. (Figure 7, Table 4). EGFR promoter methylation analysis was omitted from the 3 metastatic lesions of case #25, which showed high EGFR mRNA and protein expression due to an EGFR gene amplification.

**Table 4:** Molecular alterations of EGFR at the DNA, RNA and protein levels

Case ID	Tissue specimen	EGFR methylation (%)	EGFR mRNA expression	EGFR protein expression
**1**	NO	8.3	n.a.	0
	PT	12.7	0.008	0
	M(HEP)	20.0	0.000	0
	M(HEP)	11.3	0.147	0
**2**	NO	1.3	n.a.	0
	PT	3.0	4.733	0
	ReT	11.3	1.402	0
**3**	NO	1.7	n.a.	0
	PT	7.7	0.108	0
	M(HEP)	6.7	1.799	0
**4**	NO	1.0	n.a.	0
	PT	18.0	0.578	0
**5**	NO	9.3	n.a.	0
	**PT**	**38.7**	**0.314**	**0**
**6**	NO	1.0	n.a.	0
	PT	9.7	0.215	0
**7**	NO	9.3	n.a.	0
	PT	46.0	1.096	0
**8**	NO	10.0	n.a.	0
	**PT**	**12.0**	**2.411**	**0**
	M(HEP)	14.3	0.089	0
**9**	NO	3.3	n.a.	0
	PT	9.3	0.058	0
	M(PUL)	9.7	0.880	0
	**M(PUL)**	**16.7**	**1.722**	**1**
	M(HEP)	9.7	0.864	0
	M(PUL)	6.3	1.909	0
**10**	NO	7.7	n.a.	0
	PT	17.3	0.822	0
**11**	NO	9.0	n.a.	0
	PT	29.3	1.197	0
**12**	NO	5.0	n.a.	0
	PT	8.3	2.596	0
	M(HEP)	20.3	4.708	0
**13**	NO	5.0	n.a.	0
	PT	12.7	0.914	0
	M(HEP)	12.7	n.d	n.d
**14**	NO	2.0	n.a.	0
	PT	64.3	1.883	0
	M(HEP)	13.7	n.d	n.d
	PT	19.3	0.437	0
**15**	NO	1.7	n.a.	0
	reT	21.3	0.520	0
	M(HEP)	33.3	0.379	0
**16**	NO	5.3	n.a.	0
	PT	41.0	0.559	1
	M(HEP)	56.3	0.884	0
	M(HEP)	7.0	1.015	0
**17**	NO	1.7	n.a.	0
	PT	50.0	0.095	0
	M(HEP)	55.7	0.271	0
**18**	NO	19.3	n.a.	0
	PT	15.7	0.245	0
**19**	NO	5.3	n.a.	0
	ReT	32.0	0.691	0
**20**	NO	2.3	n.a.	0
	PT	18.7	0.224	0
	M(HEP)	8.7	0.147	0
	M(HEP)	8.7	0.047	0
**21**	NO	3.3	n.a.	0
	PT	27.3	8.039	0
**22**	NO	3.0	n.a.	0
	PT	48.3	0.520	0
**23**	NO	15.3	n.a.	0
	PT	12.3	0.079	0
**24**	NO	0.3	n.a.	0
	PT	20.7	0.467	0
**25**	NO	11.3	n.a.	0
	**PT**	**27.3**	**78.124**	**3**
	M(HEP)	n.d.	127.129	1
	M(PUL)	n.d.	50.586	1
	M(PUL)	n.d.	87.781	3

The table provides the summary of findings of EGFR promoter methylation (mean % methylation of three CpG sites investigated), EGFR mRNA expression (relative quantification qRT-PCR according to 2^-ΔΔCT method [71]) and EGFR protein expression (immunohistochemical scores [63]) analyses. N.a. = not applicable; n.d. = not determined. See text for details. Refer to Figure 7 for representative stainings (cases marked in bold in the table).

As assessed by pyrosequencing of 3 CpG sites, EGFR promoter methylation ranged from 3% to 64.3% in primary tumors and from 1.3% to 56.3% in metastases (Table 4).

The relative EGFR mRNA expression was generally low upon comparison to a universal reference RNA (fold change primary/recurrent tumors: 0.008 to 8.039; fold change liver or lung metastases: 0 to 4.708). One case (#25, gene amplification) showed a 78-fold increase of EGFR mRNA expression in the primary tumor and an up to 127-fold increase in the metastasis (Table 4).

Finally, EGFR protein expression was positive (score >/=1) in 3/25 (12%) cases (#9, #16, #25). Case #9 only showed an EGFR expression of score 1 in one lung metastasis; case #16 only showed an EGFR expression of score 1 in the primary tumor; case #25 showed an EGFR expression of score 3 for the primary tumor and one lung metastasis (Figure 7). Further to this, EGFR expression was heterogeneous resulting in a score of 0, but showing focal EGFR membranous positive tumor cells even within the same tumor glands (Figure 7, cases #8, #9).

Hence, indeed CRC rarely express high levels of membranous EGFR protein, which are not directly regulated by EGFR promoter methylation and mRNA expression (Supplementary Figure 4).

### E-cadherin protein expression correlates to response to EGFR-targeted therapy in CRC patients

**Figure 8:**
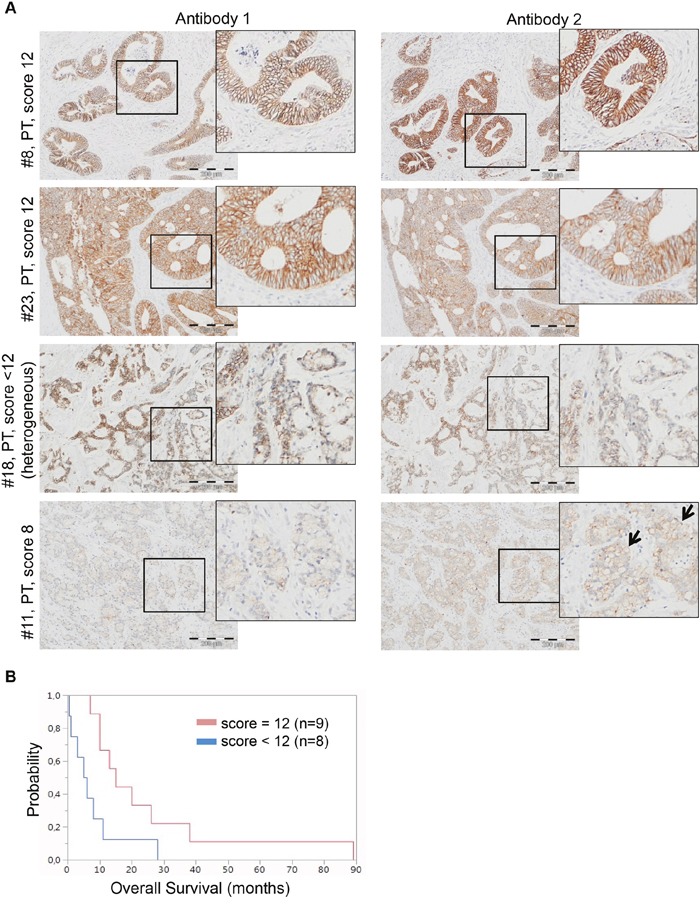
E-cadherin is differentially expressed in CRC and associates with overall survival **A**. Representative E-cadherin stainings of two responding (#8, #23) and two non-responding (#18, #11) CRC cases. Responding cases #8 and #12 both exhibit an E-cadherin IRS score of 12. Non-responding cases #18 and #11 exhibit an E-cadherin IRS score of <12 with heterogeneity and an E-cadherin IRS score of 8, respectively. Note that the two antibodies used for immunohistochemistry (antibody 1: clone NCH-38, DakoCytomation/Agilent, detects the 120kDa mature and a 82kDa soluble E-cadherin; and antibody 2= clone 24E10, Cell Signaling Technology, raised against P780 of E-cadherin) show similar expression patterns, except a slightly more prominent membranous E-cadherin expression for antibody 2 in case #11. Scoring was performed according to Kang et al. [23]. Bar= 200μm. **B**. Kaplan-Meier curves for median overall survival (OS) of colorectal cancer cases with IRS score <12 (blue line, n=8) or IRS score=12 (red line, n=9). Median OS rate of patients with IRS score <12 was 7.8 months and 25.3 months for patients with IRS score=12 (p=0.026).

To investigate whether or not other cellular mechanisms involved in CRC metastasis may predict response to EGFR-targeted therapy [24], we stained 24 primary tumors, 3 recurrent tumors and associated 18 metastases for E-cadherin protein expression (Figure 8, Table 5). As for the *in vitro* experiments above, this was performed with two commercially available antibodies: The first antibody (clone NCH-38, DakoCytomation/Agilent) recognizes the 120 kD mature form and a 82kD (soluble) fragment of E-cadherin. The second antibody (clone 24E10, Cell Signaling Technology) recognizes P780 of E-cadherin and stains cytoplasmic E-cadherin.

**Table 5:** E-cadherin protein expression in CRC

Case ID	Tissue specimen	E-cadherin protein expression		
		% positive tumor cells	intensity	IRS
**1**	NO	10-50%	weak	2
	PT	>80%	strong	12
	M(HEP)	>80%	strong	12
	M(HEP)	>80%	strong	12
**2**	NO	10-50%	moderate	4
	PT	>80%	moderate	8
	ReT	>80%	moderate	8
**3**	NO	>80%	strong	12
	PT	>80%	strong	12
	M(HEP)	>80%	strong	12
**4**	NO	10-50%	moderate	4
	PT	>80%	strong	12
**5**	NO	>80%	strong	12
	PT	>80%	strong	12
**6**	NO	>80%	strong	12
	PT	>80%	strong	12
**7**	NO	10-50%	strong	6
	PT	>80%	strong	12
**8**	NO	>80%	strong	12
	PT	>80%	strong	12
	M(HEP)	>80%	strong	12
**9**	NO	>80%	moderate	8
	PT	>80%	strong	12
	M(PUL)	>80%	strong	12
	M(PUL)	>80%	strong	12
	M(HEP)	>80%	strong	12
	M(PUL)	>80%	strong	12
**10**	NO	10-50%	moderate	4
	PT	>80%	moderate	8
**11**	NO	>80%	strong	12
	PT	>80%	moderate	8
**12**	NO	>80%	strong	12
	PT	>80%	strong	12
	M(HEP)	>80%	strong	12
**13**	NO	>80%	weak	4
	PT	>80%	strong	12
	M(HEP)			
**14**	NO	10-50%	strong	6
	PT	>80%	moderate	8
	M(HEP)			
	PT	51-80%	strong	9
**15**	NO	>80%	moderate	8
	ReT	>80%	moderate	8
	M(HEP)	51-80%	strong	9
**16**	NO	>80%	strong	12
	PT	>80%	strong	12
	M(HEP)	51-80%	strong	9
	M(HEP)	>80%	strong	12
**17**	NO	10-50%	moderate	4
	PT	>80%	moderate	8
	M(HEP)	>80%	strong	12
**18**	NO	>80%	strong	12
	**PT**	**>80%**	**heterogeneous**	**<12**
**19**	NO	>80%	strong	12
	ReT	>80%	strong	12
**20**	NO	>80%	strong	12
	**PT**	**51-80%**	**strong**	**9**
	M(HEP)	>80%	strong	12
	**M(HEP)**	**>80%**	**strong**	**12**
**21**	NO	>80%	strong	12
	PT	>80%	moderate	8
**22**	NO	>80%	strong	12
	**PT**	**51-80%**	**moderate**	**6**
**23**	NO	>80%	strong	12
	**PT**	**>80%**	**strong**	**12**
**24**	NO	>80%	strong	12
	PT	>80%	strong	12
**25**	NO	>80%	strong	12
	PT	>80%	strong	12
	M(HEP)	>80%	strong	12
	M(PUL)	>80%	strong	12
	M(PUL)	>80%	strong	12

The table provides results of immunohistochemical analysis of E-cadherin protein expression. See text for details. Refer to Figure 8 for representative stainings (cases marked in bold in the table).

Immunohistochemical analyses revealed that E-cadherin was expressed in all 25 CRC cases, with E-cadherin expression in normal epithelia being high (IRS score 12) in 15/25 cases and low (IRS score range 2 to 8) in 10/25 cases. E-cadherin protein expression was mostly maintained in case-matched primary tumors and metastases, except in three cases (#16, #17, #20) with either higher (#17, #20) or lower (#16) IRS scores for E-cadherin expression in metastases. Indeed, in 4/25 cases, primary tumors (#15, #20, #22) and liver (#15, #16) metastases showed heterogeneous E-cadherin expression as defined by the number of positive tumor cells (i.e. scored for category “51-80%” positivity) (Table 5). There was no case with complete E-cadherin negativity and both used antibodies showed similar expression patterns (Figure 8A). Nevertheless, in a single case with low E-cadherin expression (#11, primary tumor), membranous staining appeared to be more prominent by antibody 2 as compared to antibody 1 (Figure 8A). Since entire tissue specimen sections were used for immunohistochemical staining, artefacts due to non-representative selected tissue areas can be ruled out.

Finally, upon classification of primary tumors of cases with clinical follow-up (n=17) into “E-cadherin score <12” (n=8) versus “E-cadherin score=12” (n=9), the median overall survival of E-cadherin strong positive cases was better than in those cases with lower E-cadherin expression (Figure 8B).

### A matrix for response prediction to EGFR-targeted therapy in CRC

Of the generated data, RAS status, BRAF, PIK3CA, ATM mutations as well as E-cadherin expression appeared to correlate with responses of CRC cell lines and cases to EGFR-targeted therapy. In order to provide a synopsis of their predictive value, CRC cell lines and cases were defined as responders, intermediate responders and non-responders followed by integration of the key alterations investigated. The classification was by the ADCC data for cell lines (Figure 1) and by known clinical response parameters of “partial/complete response”, “stable/progressive disease” and survival data for CRC cases (Table 2).

As depicted in Table 6 for primary tumors, the MSI and CIMP status as well as EGFR expression are not of predictive value in the present cohort. Similarly, RAS mutations were actually present in responders, intermediate responders and non-responders. The same is true for BRAF and PIK3CA mutations in general. However, when specifying PIK3CA mutations, the responding cell line (DLD1) and CRC patients (#3, #8) harbored the same PIK3CA exon 9 mutations, whereas intermediate responders and non-responders carried the same PIK3CA exon 20 mutations (HCT116, LS174T, RKO cell lines, and recurrent tumor, primary tumor or metastases of CRC cases #2, #14, #16). In contrast, ATM mutations and E-cadherin expression appear to stratify responders (ATM wildtype sequences and high E-cadherin expression) from intermediate responders and non-responders (ATM mutations and low E-cadherin expression).

**Table 6:** A matrix for response prediction to EGFR-targeted therapy in CRC

EGFR-targeted Tx response	ID#	MSI	CIMP	RAS	BRAF	PIK3CA	ATM	EGFR protein	E-cadherin protein
**Responders**	DLD1	+	+	mut.	**wt**	mut.	**wt**	(+)	**+++**
	3	-	-	**wt**	**wt**	mut.	**wt**	0	**12**
	8	-	-	**wt**	**wt**	mut.	**wt**	0	**12**
	15	-	-	**wt**	**wt**	**wt**	**wt**	0	9
	1	-	-	mut.	**wt**	**wt**	**wt**	0	**12**
	23	-	-	mut.	**wt**	**wt**	**wt**	0	**12**
	9	-	-	**wt**	**wt**	**wt**	**wt**	0	**12**
	6	-	-	**wt**	**wt**	**wt**	**wt**	0	**12**
**Intermediate responders**	HCT116	+	+	mut.	**wt**	mut.	**wt**	(+)	+
	LS174T	+	-	mut.	**wt**	mut.	**wt**	++	++
	Caco-2	-	-	**wt**	**wt**	**wt**	**wt**	+++	+
	HT29	-	+	**wt**	mut.	**wt**	mut.	++	++
	17	-	n.d.	**wt**	**wt**	**wt**	mut.	0	8
	13	-	-	**wt**	**wt**	**wt**	wt (mut.)	0	**12**
	4	-	-	**wt**	**wt**	**wt**	**wt**	0	**12**
	10	-	-	**wt**	**wt**	**wt**	**wt**	0	8
	16	-	-	mut.	**wt**	wt (mut.)	wt (mut.)	1	**12**
**Non responders**	SW480	-	-	mut.	**wt**	**wt**	**wt**	-	(+)
	RKO	+	+	**wt**	mut.	mut.	mut.	-	(+)
	14	-	-	mut.	**wt**	wt (mut.)	**wt**	0	8
	18	+	+	**wt**	mut.	**wt**	**wt**	0	<12 heterogeneous
	21	-	-	mut.	**wt**	**wt**	**wt**	0	8
	2	-	-	mut.	**wt**	wt (mut.)	wt (mut.)	0	8
	11	-	-	**wt**	**wt**	**wt**	**wt**	0	8
**unknown**	5	-	-	**wt**	**wt**	**wt**	**wt**	0	**12**
	7	-	-	mut.	**wt**	**wt**	**wt**	0	**12**
	12	-	-	mut.	**wt**	mut.	**wt**	0	**12**
	19	-	+	**wt**	**wt**	**wt**	**wt**	0	**12**
	20	-	-	**wt**	**wt**	**wt**	**wt**	0	9
	22	-	-	mut.	**wt**	**wt**	**wt**	0	**12**
	24	-	-	**wt**	**wt**	**wt**	**wt**	0	**12**
	25	-	-	**wt**	**wt**	**wt**	mut.	3	**12**

The table shows alterations of primary tumors (and in recurrent tumors or metastases, parentheses). Responders >12 months overall survival, intermediate responders >6 to 12 months overall survival, non-responders =/<6 months overall survival. mut =mutated, wt=wildtype; n.d.= not determined; “+” = positive; “-” = negative.

## DISCUSSION

Treatment of metastatic colorectal cancer (CRC) patients may include EGFR-targeted therapy if the tumor does not harbor an activating downstream RAS mutation. The monoclonal anti-EGFR antibody (Cetuximab, Panitumumab) based therapies are hence considered to be effective even without prior detection of EGFR protein expression [34]. Still, even RAS mutated CRCs may be responsive and vice-versa also RAS wildtype CRCs may be resistant to EGFR-targeted therapy. To elucidate potential mechanisms underlying treatment responses to EGFR-targeted therapy, we here comprehensively investigated 7 CRC cell lines and tissue specimens of 25 CRC patients for putative resistance mechanisms residing within the target (i.e. EGFR), mutations of downstream signaling pathways or in bypass receptor tyrosine kinases as well as E-cadherin expression. Indeed, ATM and PIK3CA mutations as well as E-cadherin expression may represent supplementary predictive markers for response to EGFR-targeted therapy in CRC cell lines and patients.

As reported before [35–38], EGFR mRNA and protein expression was low or undetectable in most of the CRC cell lines and tissue specimens studied. If present, EGFR protein expression was non-membranous. Only one CRC case showed EGFR overexpression, most likely explained by gene amplification [35], however this was not related to EGFR-treatment response.

In CRC cell lines low EGFR expression correlated to EGFR promoter methylation, but this was not the case for CRC tissue specimens. A similar discrepancy of EGFR promoter methylation and expression in CRC tissue specimens was previously reported by Scartozzi et al. [16] and Chiadini et al. [17]. In view of this, so far EGFR methylation status [16, 17] or even genome-wide methylation data [39] is controversially discussed as a predictive marker for EGFR-targeted therapy.

Moreover, surprisingly EGFR protein expression itself failed as predictive marker to EGFR-targeted antibody-based therapy responses: For example, CRC patients may respond to Cetuximab even without EGFR expression [34] and - vice versa – CRC patients with EGFR-positive tumors do not exhibit higher response rates as compared to those CRC patients with EGFR-negative tumors [40, 41]. Indeed, both our *in vitro* and *in situ* analyses show that EGFR lacking tumor cells (e.g. DLD1) may be responsive to EGFR inhibition. An explanation for the positive response to EGFR-targeted inhibition in EGFR-negative tumor cells *in vitro* or tumor tissue specimens may be due to the dynamic nature of receptor tyrosine kinases protein stability, shuttling between cytosol and membrane as well as membranous anchoring and cleavage. Hence, we confirm previous studies in that EGFR (over)expression is not a feasible predictive marker for EGFR-targeted therapy, quite in contrast to what is seen for e.g. HER2-targeted therapy in breast cancers [42].

Furthermore, we did not detect the recently reported EGFR “resistance” mutations in EGFR exon 12 (p.G465 or p.S492). However, these were reported to be absent in KRAS wildtype tumors prior to EGFR-targeted therapy [43, 44] and to emerge during EGFR-targeted treatment [30, 33]. This underlines our finding of “baseline” EGFR exon 12 wildtype sequences. Interestingly, 7 of 25 CRC cases carried a known EGFR SNP in intron 12/13 (rs1558544) but this did not correlate with EGFR-targeted treatment response. The EGFR protein structure of this EGFR variant remains unchanged according to AASsites [45] and has been detected before in Gefitinib treated lung adenocarcinoma patients without an association with survival [46].

Thus, by next investigating the current predictive marker RAS mutation status by targeted next generation sequencing (tNGS), we identified RAS mutations in CRC cell lines and tissue specimens of CRC patients treated by EGFR-targeted therapy. In fact, RAS mutations were seen in both responding and non-responding CRC cell lines as well as in CRC cases with long and short overall survival. The responding cell line DLD1 and the intermediate responding cell line HCT116 both carried a KRAS codon p.G13 mutation, which was in fact reported previously as being a “beneficial” mutation associated with Cetuximab response by others before [47–49]. This RAS mutation was not detected in the CRC cases. The CRC cases mostly harbored a RAS exon 3 codon p.Q61 mutation, which at the time of treatment of this retrospective CRC cohort was not yet included into routine diagnostic and clinical guidelines. Of the 8/25 patients with RAS mutations 5/8 showed progressive disease, for 2/8 no information about response rate was available, further underlying the concept of comprehensive RAS testing [7, 50]. However, RAS testing alone cannot explain the poor response in other CRC cases.

Hence, by tNGS we next screened for alterations in genes coding for receptor tyrosine kinases, RAS/MAPK and PIK3/AKT signaling pathways, which may bypass EGFR inhibition by antibody-based therapies. We detected alterations in RTK up to 59.6%, RAS/MAPK in up to 38.3% and PIK3/AKT in up to 27.7%. No direct correlation was found between these mutations and response to EGFR-targeted therapy as evaluated by overall survival. Indeed, combination of mutations in these down-stream signaling pathways were shown to influence direct (B)RAF inhibition [51, 52] rather than EGFR-targeted therapy.

Of interest were the detected PIK3CA mutation patterns. PIK3CA is frequently mutated in several solid tumors [8] and mostly occur in PIK3CA exon 9 within the coding region for the helical domain and exon 20 within the coding region for the kinase domain [53], which was also the case in our study (Supplementary Figure 4). The PIK3CA exon 9 p.E545K as such leads to a change in charge of the protein [53], whereas the PIK3CA p.H1047R mutations results in constitutive activation [54]. Whilst PIK3CA exon 9 mutation may be without gross functional consequences, the activating exon 20 mutation may result in resistance to up-stream EGFR-targeted therapy. Indeed, the CRC cell line DLD1 responding to Cetuximab exhibited two PIK3CA exon 9 mutations (p.E545K, p.D549N), PIK3CA exon 20 mutations (p.H1047R) were only observed in CRC cases with progressive disease and short overall survival under EGFR-targeted therapy. A direct predictive value of PIK3CA mutations for Cetuximab resistance is still controversially discussed [55, 56], but PIK3CA mutations were reported by others to e.g. also significantly correlate with lower progression free and overall survival of CRC patients [7].

Besides direct EGFR signaling associated genes, the most frequent mutations observed in CRC cell lines and cases were ATM, SMAD4, KDR, ErBB4 and FBXW7, as reported by others for CRC before [57, 58]. Indeed, SMAD4, ErBB4 and FBXW7 mutations appear to confer treatment resistance to Cetuximab [5]. However, this association was not seen in our study, with e.g. SMAD4 (59), ErBB4 (39) and FBXW7 (38) mutations being present in both EGFR-targeted therapy responders and non-responders. In contrast, our study rather suggests ATM mutations to be involved in treatment resistance to Cetuximab. ATM (ataxia telangiectasia mutated) belongs to the PI3/PI4-kinase family, is able to form a complex with EGFR [58], causes phosphorylation of AKT [59] and was suggested as a therapeutic target in cancer [12]. The possible functional consequences of the here detected ATM mutations in the EGFR pathway still need to be elucidated, but are beyond the scope of this study.

Irrespective of the comprehensive analyses of EGFR and mutations in associated signaling pathways, other mechanisms may influence EGFR functionality and its inhibition by therapeutic agents. As such, E-cadherin plays a role in EGF receptor recruitment and activation [59, 60]. Moreover, it was shown that cells loosing E-cadherin are able to circumvent the classical EGFR signaling and therefore acquire resistance to treatment with antibodies against EGFR [24]. Finally, restoring E-cadherin expression is postulated to increase the sensitivity to anti-EGFR treatment [61]. Indeed, in our *in vitro* experiments down-regulation of E-cadherin by siRNA in DLD1 cells (high E-cadherin expressing cells, Cetuximab responders) abrogated their response to Cetuximab. Moreover, two of the non-responding CRC cell lines had only weak and/or cytoplasmic E-cadherin expression, lacking the mature E-cadherin protein. One hypothesis of how this E-cadherin driven resistance mechanism may act, is that E-cadherin is shed from the tumor cells in a soluble form, which – as shown in *in vitro* model systems [22] – may then activate EGFR promoting cell survival [62]. Our present data does not directly confirm that E-cadherin cleavage and presence of elevated levels of soluble E-cadherin are responsible for Cetuximab resistance, but it clearly shows that dynamic regulation of E-cadherin expression and intracellular localization, respective E-cadherin maturity does have an impact on CRC cells responses to Cetuximab. This was also seen *in situ*, were we found 9/25 CRC cases which lacked strong and complete membranous E-cadherin expression in tumor cells. This is in part in accordance with a recent study [23], which investigated 229 not therapy-preselected CRC patients and only detected 4.3% of cases being E-cadherin negative. Examining the precise dynamic nature of E-cadherin expression and subcellular localization and/or its cleavage and soluble form in formalin-fixed and paraffin-embedded tissue specimens is currently technically not possible. Nevertheless, our data clearly demonstrates that a loss of E-cadherin expression is linked to EGFR-targeted therapy non-responder CRC cell lines and cases.

In summary, our study comprehensively analyses both CRC cell lines and tissue specimens for known and novel putative predictive markers of EGFR-targeted therapy responses and is the first to identify ATM mutations and E-cadherin expression as potential novel supportive predictive markers for EGFR-targeted therapy.

## MATERIALS AND METHODS

### Cell lines

All cell lines (Caco-2, HT29, SW480, DLD1, HCT116, LS174T, RKO) were cultured in a humidified atmosphere (37°C, 5% CO_2_) in their corresponding medium supplemented with 10% fetal calf serum (Life Technologies) and 1% Glutamin (GE Healthcare). Caco-2, LS174T, RKO and SW480 were cultured in DMEM, high glucose, DLD1 in RPMI, HCT116 and HT29 in McCoys 5A medium (all Life Technologies). All CRC cell lines were screened for mycoplasma contamination using Venor GeM Classic Mycoplasma Detection Kit (Minerva biolabs) and DNA fingerprinting was performed for all seven cell lines at the Leibniz Institute DSMZ, Braunschweig, Germany.

### Cell viability assay

The effect of Cetuximab (MerckSerono) on cell proliferation of colorectal cancer cell lines (Caco-2, HT29, SW480, DLD1, HCT116, LS174T, RKO) was measured using Cell titer 96® Aqueous One Solution Cell Proliferation Assay (MTS) (Promega). 2.0 – 3.5x10^3^ cells/well were seeded in triplicates on a 96 well plate and allowed to adhere over night at 37°C in a 5% CO_2_ humidified atmosphere in their corresponding medium. The cells were treated with Cetuximab (0.1, 1, 10, 50, 100 μg/ml) and 0.9% NaCl solution as control for 72h. Then, 20μl of MTS solution was added and the absorbance was measured at 490nm with a microplate reader (Tecan) after additional 2h incubation at 37°C. Proliferation was recorded as % of control.

### Antibody-dependent cellular cytotoxicity (ADCC)

The antibody-dependent cellular cytotoxicity assay was performed according to Fichter et al. [63]. Peripheral blood mononuclear cells (PBMCs) were isolated using Pancoll lymphocyte separation medium (PAN-Biotech GmbH). PBMCs were incubated with target cells (Caco-2, HT29, SW480, DLD1, HCT116, LS174T, RKO) at various effector-to-target ratios (1:1, 1:2.5, 1:5, 1:10, 1:20, 1:40) in triplicates in medium alone or in medium in the presence of 10μg/ml Cetuximab (MerckSerono). After 4h, cytotoxicity was measured using the CytoTox 96® Non-Radioactive Cytotoxicity Assay (Promega). The absorbance was recorded at 490nm using a 96-well plate reader (Tecan). Percentage of cytotoxicity was calculated using the following equation: %cytotoxicity = (experimental - effector spontaneous - target spontaneous) / (target maximum - target spontaneous) x 100.

### Down-regulation of E-cadherin by siRNA

As adapted from the siRNA approach used by us before [64] 1.7x10^5^ DLD1 cells were transfected with either 100nM E-cadherin siRNA (SMARTpool: si Genome CDH1siRNA, GE Dharmacon) or Silencer negative control siRNA, Thermo Fisher Scientific using Dharma Fect2 Transfection Reagent, GE Dharmacon. Transfection control was with DharmaFECT 2 Transfektion Reagent, GE Dharmacon.

Cells were investigated at 72h post siRNA treatment for E-cadherin protein levels by Western Blot and were subjected to ADCC at 72h post siRNA treatment.

### Immunofluorescence staining of colorectal cancer cells

3x10^4^cells/well were seeded onto a 24 well plate, covered with sterile coverslides and allowed to adhere overnight in their corresponding medium. Cells were fixed in 2% formalin for 20 min at room temperature (RT), permeabilized for 10 min at 4°C in 0.5% Triton-X100/PBS, blocked for 1h at RT in 5% normal goat serum/0.3% Triton-X100/PBS. E-cadherin antibody (1:200, clone 24E10, Cell signaling) and normal rabbit IgG isotype control (Cell signaling) were incubated overnight at 4°C. After removing the antibodies and washing with 1x PBS the secondary antibody goat anti-rabbit IgG A488 (1:200) was added and incubated for 1h in the dark. The cells were washed again and were counterstained with vectashield mounting medium with DAPI (vector laboratories). Pictures were taken using a fluorescence microscope (Axioplan 2 imaging with apotome system, Carl Zeiss).

### Cell lysis and western blotting

Total protein lysates were prepared using Qproteome Mammalian Protein Prep Kit (Qiagen). The following antibodies were used for immunodetection: E-cadherin (1:2000, clone 24E10, Cell signaling), β-Actin (1:5000, Sigma Aldrich). Bands were quantified by using fusion capt Advance FX7 software (Vilber).

### Detection of soluble E-cadherin by ELISA

Soluble E-cadherin was measured in cell culture supernatants using Quantikine ELISA (human E-cadherin immunoassay, R&D Systems) according to the manufacturer′s instructions. Cell culture supernatants of seven colorectal cancer cell lines were harvested after 48h and 72h. Three independent experiments were performed and data given as mean +/- standard deviation.

### Patients and tissue specimens

This study included 25 colorectal cancer patients, of whom primary or recurrent tumors as well as liver or lung metastases in 12/25 of cases were examined. 23/25 CRC cases were previously tested for KRAS exon 2 mutations at the Institute of Surgical Pathology, Medical Center – University of Freiburg, Freiburg, Germany. All patients were treated with Cetuximab and/or Panitumumab, 22/25 received concurrent chemotherapy. All patients underwent surgery at the Department of Surgery, Medical Center – University of Freiburg, Freiburg, Germany between 2002 and 2014.

Tissue specimens were formalin-fixed and paraffin-embedded (FFPE). Normal colonic epithelial cells were derived from tissue blocks of the resection margin, whereas primary tumors and metastases were derived from central tumor mass. The use of tissue specimens was approved by the local ethics institution (#251/04/07/09, #66/07 and #191/13 Ethik-Kommission, University of Freiburg, Germany). The clinico-pathological characteristics of the colorectal cancer patients are summarized in Table 2.

### Immunohistochemistry staining

Serial sections (2μM) of formalin-fixed and paraffin-embedded (FFPE) tissues were used for EGFR and E-cadherin staining. The tissue sections were incubated at 37°C (EGFR) or 58°C (E-cadherin) overnight, deparaffinized with xylene and rehydrated with ethanol in decreasing concentrations (96%, 70%). After antigen retrieval (EGFR: digestion with 0.05% proteinase K for 5min; E-cadherin NCH-38: pH6.1 buffer by pressure cooker/sico for 2min; E-cadherin 24E10: pH6.1 buffer, pressure cooker for 5 min), tissue sections were stained with diluted antibodies to EGFR (mouse monoclonal, clone H11, 1:500, 1 hr, DakoCytomation/Agilent) and to two different antibodies E-cadherin (mouse monoclonal, clone NCH-38, 1:1000, 30min, DakoCytomation/Agilent; rabbit monoclonal, clone 24E10, 1:100, 30min, Cell Signaling Technology,) followed by the Dako REAL Detection System (alkaline phosphatase/RED, rabbit/mouse, Dako) for EGFR and the EnVision^TM^ Flex System for E-cadherin.

All steps were performed on an autostainer (Dako). Scoring for EGFR was according to Fichter et al. [63]. Score 0=negative expression/incomplete membranous in ≤10% tumor cells, score 1=partial membranous expression in >10%tumor cells, score 2=weak, but complete membranous expression in >10% tumor cells and score 3=strong and complete membranous expression in >10% tumor cells. Scoring for E-cadherin was performed according to Kang et al. [23], with an immunoreactive score (IRS) that combines % immunopositive cells (0=0%, 1=<10%, 2=10-50%, 3=51-80%, 4>80%) with staining intensity (0=no staining, 1=weak, 2=moderate, 3=strong) resulting in overall IRS scores from 0-12. IHC stainings were evaluated by two independend observers (ALG, LL).

### Microdissection, DNA and RNA isolation

Tumor cells were marked for all tissue specimens on newly prepared hematoxylin-eosin sections by a qualified pathologist (LL) for subsequent microdissection of tumor cells under morphological control. Tissue slides were deparaffinized with xylene and rehydrated with ethanol in decreasing concentrations (100%, 90%, 70%, 50%), briefly stained in hematoxylin and digested overnight. DNA was isolated using the QIAamp DNA FFPE Tissue Kit and RNA was isolated using the RNeasy FFPE Kit. DNA and RNA of patient #25 were available from a previous isolation using the AllPrep DNA/RNA FFPE Kit. Total DNA of cell lines was isolated using DNeasy Blood &Tissue Kit and total RNA was isolated using the RNeasy Mini Kit. All isolation kits were from Qiagen and isolations were according to manufacturer′s instructions.

### Microsatellite instability testing (MSI)

For MSI testing, a new multiplex PCR protocol was established with modifications to a previous protocol [65]. In brief, 25-μl PCR multiplex fluorescent reaction mix was composed of 1 x PCR buffer (Qiagen), 2mM MgCl_2_ (Qiagen), 0.4 mM dNTPs (ThermoFisher Scientific), Taq-Polymerase 1.25 units (Qiagen) and primer sets FAM or VIC-labeled in the following concentrations: 0.1μM (Bat25-F-NED, Bat26-F-VIC, D2S123-F-VIC, D5S346F-FAM), 0.2μM (Bat25-R, Bat26-R, D2S123-R, D5S346-R, D17S250-F-FAM), 0.4μM (D17S250-R). 100ng normal and tumor DNA were used for PCR, which was performed in a Biometra Professional TRIO Thermocycler according to cycling conditions of Berg et al [65]. Fluorescently labeled PCR products were detected using ABI PRISM 3130xl Genetic Analyzer and GeneMapper 4.0 software for data analysis.

### CpG island methylator phenotype classification and analysis of EGFR promoter methylation

For determination of CpG island methylator phenotype (CIMP) and EGFR promoter methylation, DNA was bisulfite converted using the EpiTect Fast Bisulfite Conversion Kit, according to manufacturer′s instructions (Qiagen). For each gene specific PCR reaction, 20 ng of bisulfite converted DNA was used. CIMP type definition was performed according to modified protocols by Weisenberger et al. [66], Zlobec et al. [67] and Ogino et al. [68]. Methylation of five CIMP-related genes RUNX3, CACNA1G, EPM2AIP1/MLH1, NEUROG1 and CRABP1 was measured using the following PyroMark CpG assays: Hs_RUNX3_08_PM (PM00000161), Hs_CACNA1G_02_PM (PM00064736), Hs_EPM2AIP1/MLH1_01_PM (PM00104832), Hs_NEUROG1_01_PM (PM00023632) Hs_CRABP1_02_PM (PM00059605) (Qiagen). Data was analyzed using the PyroMark Q24 Software (Qiagen). Three to five CpG sites were evaluated per gene. A gene was classified as methylated, if the difference in mean methylation between tumor and the corresponding normal tissue was ≥ 19%. A case was defined as CIMP positive, if ≥ 3/5 of analyzed CIMP-related genes were methylated and CIMP negative otherwise. EGFR methylation analysis of three CpG sites was performed as above using the Hs_EGFR_02_PM (PM00030569) PyroMark CpG assay (Qiagen).

### Next generation sequencing

As reported by us before [69], DNA quality was measured using FFPE QC kit and libraries were prepared using the TruSeq Amplicon Cancer Panel (48 cancer related genes) (both Illumina). Quantity and quality of libraries were examined using the Bioanalyzer 2100 system (Agilent Technologies) and DNAs were pooled for sequencing on the MiSeq (Illumina). Mutations, passing the filter, were listed using VariantStudio (Illumina). Missense, stop gained and frameshift mutations displaying read depth >100, alt variant frequency >10 and occurring inside the gene were incorporated in the study.

### EGFR mutation analysis by dideoxy sequencing

EGFR Exon 12 mutations at codon G465 and S492 were analyzed using the following primers. G465: For-5′-TTTCTCTTGCAGTCGTCAGC-3′, rev-5′-TGCAGCTGTTTTCACCTCTG-3′. S492R: For-5′-GTGCTATGCAAATACAATAAACTGG-3′ and rev-5′GGACCCATTAGAACCAACTCC-3′ [43] 100 ng of DNA were amplified using 20μM/primer in a first PCR. The PCR products were purified using QIAquick PCR purification kit according to manufacturer′s instructions (Qiagen) and a second cycle-PCR for sequencing was performed. For dye terminator removal, DNA products were cleaned up via DyeEx 2.0 Spin Kit (Qiagen) and sequenced on an ABI 3130xl Genetic Analyzer (ThermoFisher Scientific, Waltham, United States).

### EGFR mRNA expression

EGFR mRNA expression was measured by q-RT-PCR. First, 750 ng RNA was transcribed in cDNA using M-MLV reverse transcriptase (Invitrogen, Carlsbad, United States). Next, cDNA was amplified with primers and probes for EGFR (For-5′- GCCTCCAGAGGATGTTCAATAA-3′, rev-5′-TGAGGGCAATGAGGACATAAC-3′, probe 5′-TGAG GTGGTCCTTGGGAATTTGGA-3′) and the reference gene TBP (for-5′-GCCCGAAACGCCGAATAT-3′, rev-5′-C CGTGGTTCGTGGCTCTCT-3′, probe 5′-ATCCCAAGC GGTTTGCTGCGG-3′) [70]. The analysis was performed on a 7900HT fast real time PCR system (Applied Biosystems). Relative expression of the genes of interest was calculated using the 2^-ΔΔCT^-method. [71, 72]. Besides unknown samples, a universal human reference RNA (Agilent Technologies) was transcribed in cDNA and used as reference to calculate the relative fold change of EGFR mRNA expression.

### Statistical analysis

Statistical analysis was performed using JMP version 12 statistical software. Survival distribution was estimated by the Kaplan-Meier method. Significant differences were evaluated by log-rank test and a level of 0.05 was considered statistically significant. Overall survival (OS) was defined as the interval between the start of Cetuximab/Panitumumab treatment to death.

## SUPPLEMENTARY FIGURES AND TABLES


