# Supplementary material for: ATM mutations and E-cadherin expression define sensitivity to EGFR-targeted therapy in colorectal cancer
Source: Oncotarget. 2017 Feb 9;8(10):17164–90. doi: 10.18632/oncotarget.15211 (PMC5370031; doi:10.18632/oncotarget.15211)
Supplement: Supplementary file 1 [file oncotarget-08-17164-s001.pdf]

ATM mutations and E-cadherin expression define sensitivity to EGFR-targeted therapy in colorectal cancer

SUPPLEMENTARY FIGURES AND TABLES

A

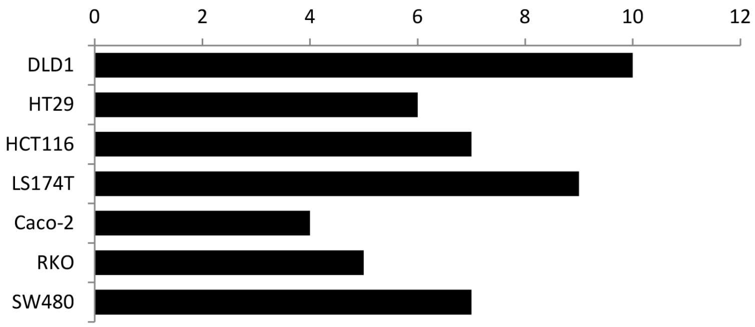

B

	TP53	APC	KDR	Kit	KRAS	PIK3CA	ATM	BRAF	HNF1A	SMAD4	SMO	ABL1	CTNNB1	IDH1	Notch1	STK11	∑ mutations
DLD1	2	1		1	1	2					1			1	1		10
HT29	1	1	1	1				1		1							6
HCT116	1		1		1	1			1		1	1					7
LS174T	1	1	1	1	1	1			1				1			1	9
Caco-2	2	1								1							4
RKO	1		1			1	1	1									5
SW480	2	1	1	1	1		1										7

C

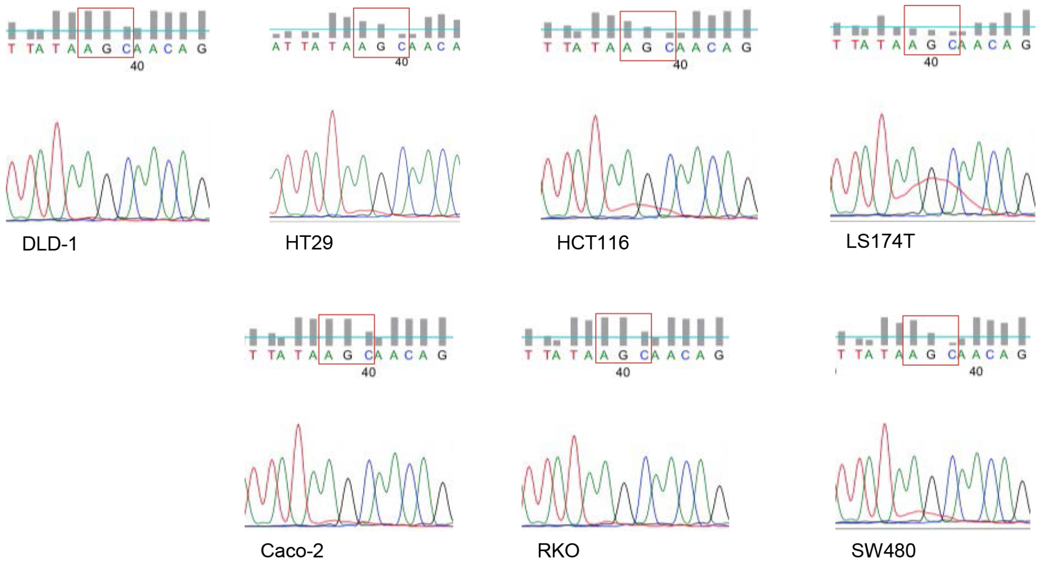

Supplementary Figure 1: Mutation profiles of CRC cell lines. A. Number of mutated genes in CRC cell lines investigated. **B.** List of mutated genes (16/48, 33.3%) and sum of mutations detected in CRC cell lines. **C.** EGFR exon 12 p.S492R mutation screening by dideoxy sequencing, showing wild type sequences in the chromatograms for seven CRC cell lines.

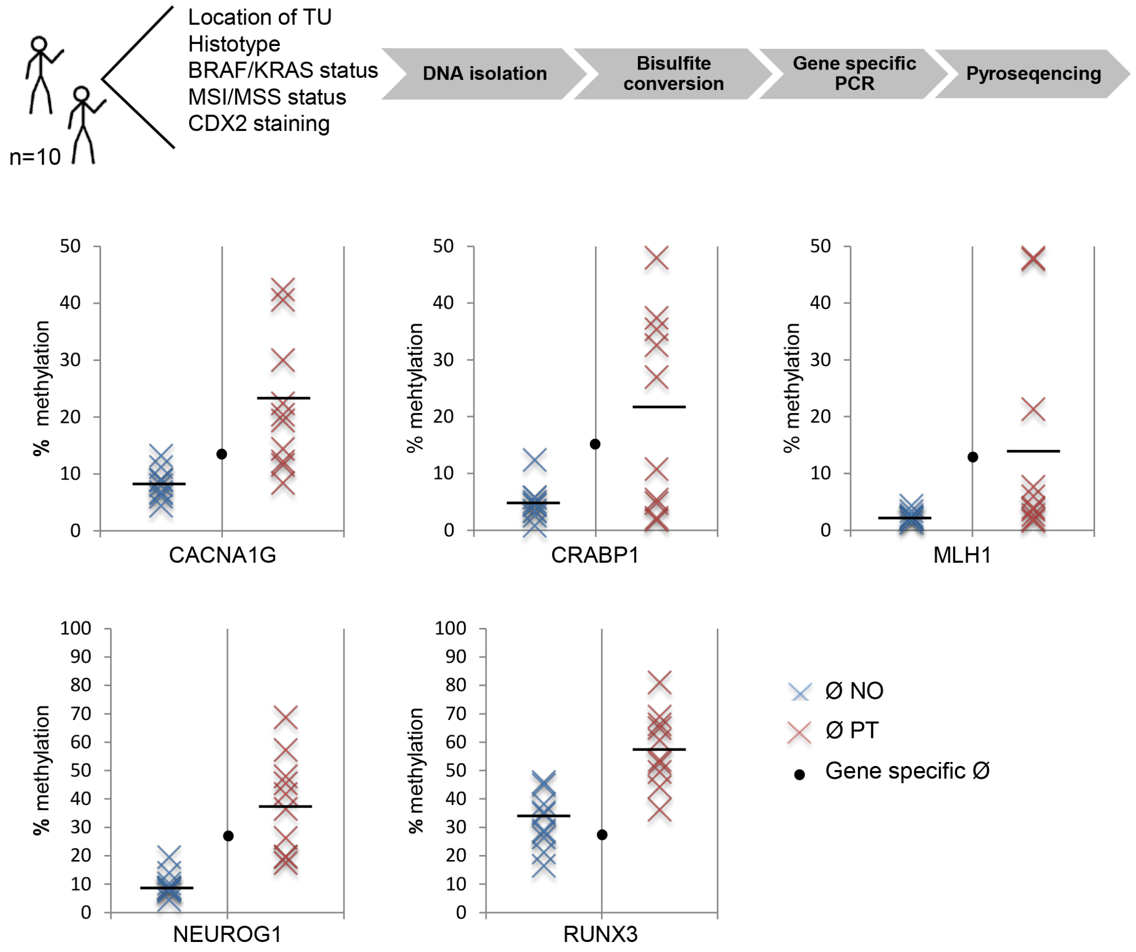

Supplementary Figure 2: Validation of CpG Island Methylator Phenotype (CIMP) analysis. Threshold for CIMP analysis was determined using a test cohort consisting of ten patients with known information about location of tumor, histotype, BRAF/KRAS status, MSI/MSS status and CDX2 staining pattern. Pyrosequencing resulted in mean % methylation of three to five CpG sites per gene (CAGNA1G, CRABP1, MLH1, NEUROG1, RUNX3) of normal (blue crosses) and primary tumor tissue (red crosses). Mean of all normal values was substrated from mean of all tumor values (black lines) and resulted in gene specific values (black dots). 13.9 for CACNA1G, 15.7 for CRABP1, 12.0 for MLH1, 28.3 for NEUROG1, 25.8 for RUNX3. Mean of all five gene specific mean values resulted in the overall threshold of ≥ 19.1%.

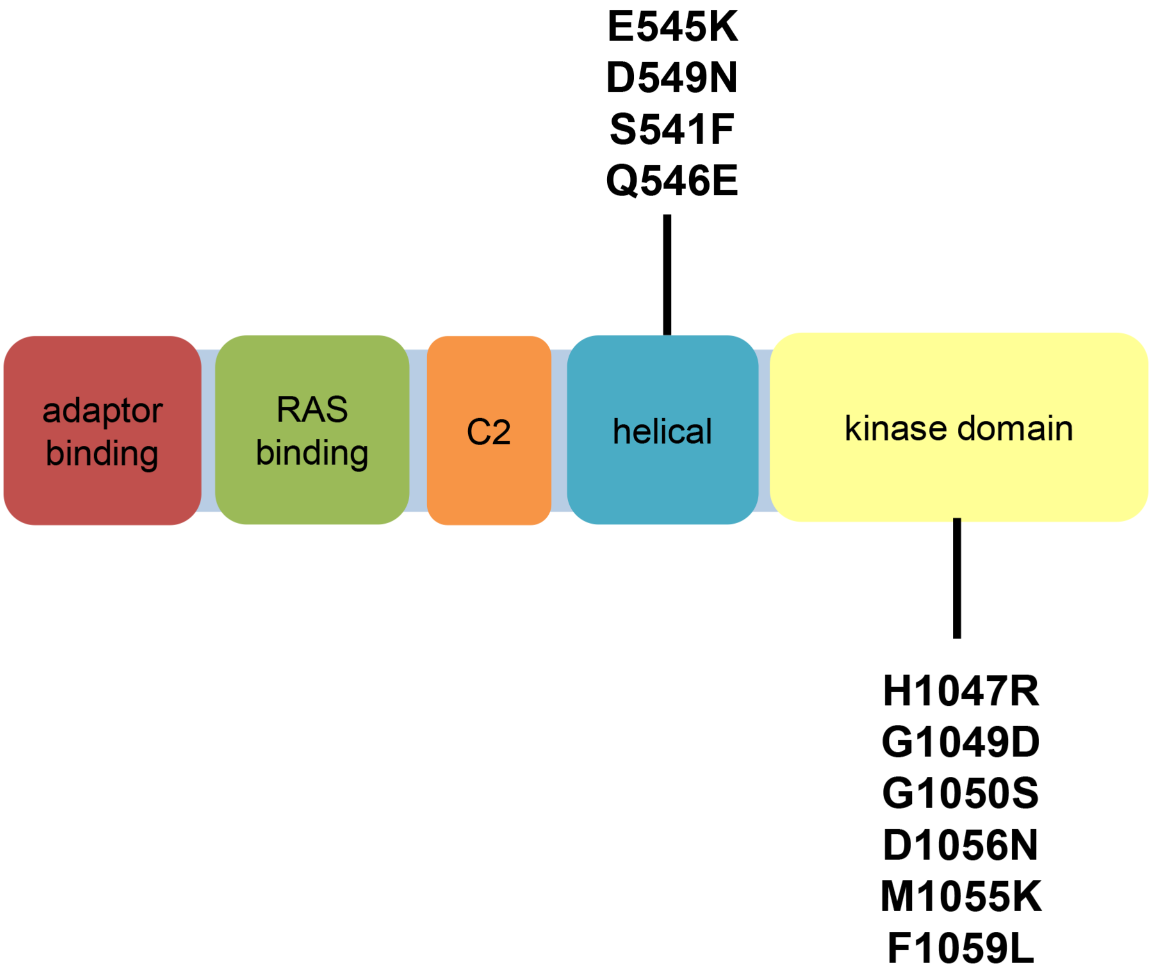

Supplementary Figure 3: Functional domains of PI3K and detected mutations. The detected PIK3CA mutations (refer to Table 1, 5 and Supplementary Table 3) are located in the helical domain (exon 9) and kinase domain (exon 20) of PI3K.

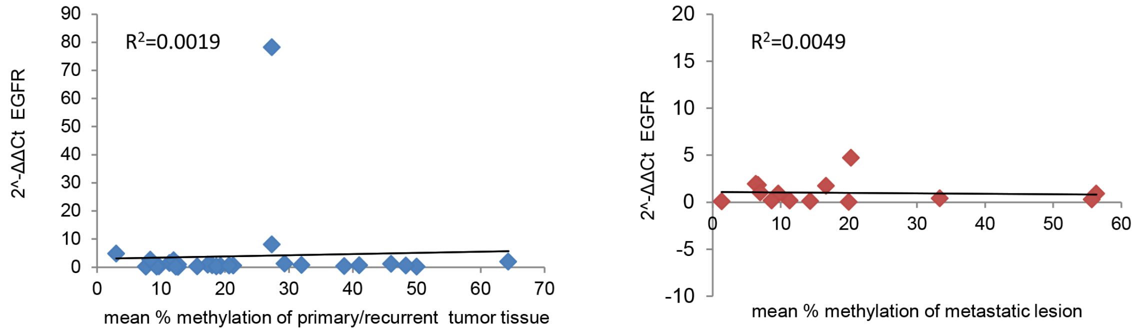

Supplementary Figure 4: Correlation of EGFR mRNA expression with EGFR promoter methylation in CRC. EGFR mRNA expression is presented as ΔCT-values and EGFR methylation as mean % for primary/recurrent tumors (left, blue) and metastases (right, red). No significant correlation is seen.

Supplementary Table 1: Summary of detected EGFR mutations

ID	TU/M	exon	codon	alt variant	consequence	domain
2	PT	19	Lys739Ile	10.7	missense	tyrosine kinase
	ReT	3	Pro136Ser	17.92	missense	x
		7	Pro296Leu	11.56	missense	ligand binding
14	PT	7	Lys294Glu	14.54	missense	ligand binding
		15	Pro622Leu	53.56	missense	ligand binding
15	PT	15	Ala611Ser	10.21	missense	ligand binding
16	M(HEP)	21	Ala859Val	29.1	missense	tyrosine kinase
	M(HEP)	3	Pro136Ser	17.02	missense	x
		15	Tyr626Phe	11.54	missense	ligand binding
		19	Leu760Phe	15.04	missense	tyrosine kinase
17	PT	15	Lys609Met	26.37	missense	ligand binding
		15	Ala611Val	18.77	missense	ligand binding
		19	Leu760Phe	29.11	missense	tyrosine kinase
		15	Thr625Ile	18.55	missense	ligand binding
20	M(HEP)	18	Leu718Met	26.27	missense	tyrosine kinase
		18	Ala722Val	14.8	missense	tyrosine kinase
25	PT	7	Gly288ValfsTer5	1.0	frameshift	ligand binding
	M(HEP)	7	Gly288ValfsTer5	1.0	frameshift	ligand binding
	M(PUL)	7	Gly288ValfsTer5	1.0	frameshift	ligand binding
	M(PUL)	7	Gly288ValfsTer5	1.0	frameshift	ligand binding

EGFR mutations detected via targeted next generation sequencing.

Supplementary Table 2: Summary of mutations detected by tNGS. Genes are listed according to frequency of detected mutations. Black coloring means detection of at least one mutation within the gene. **A** Part 1/2. **B** Part 2/2.

A

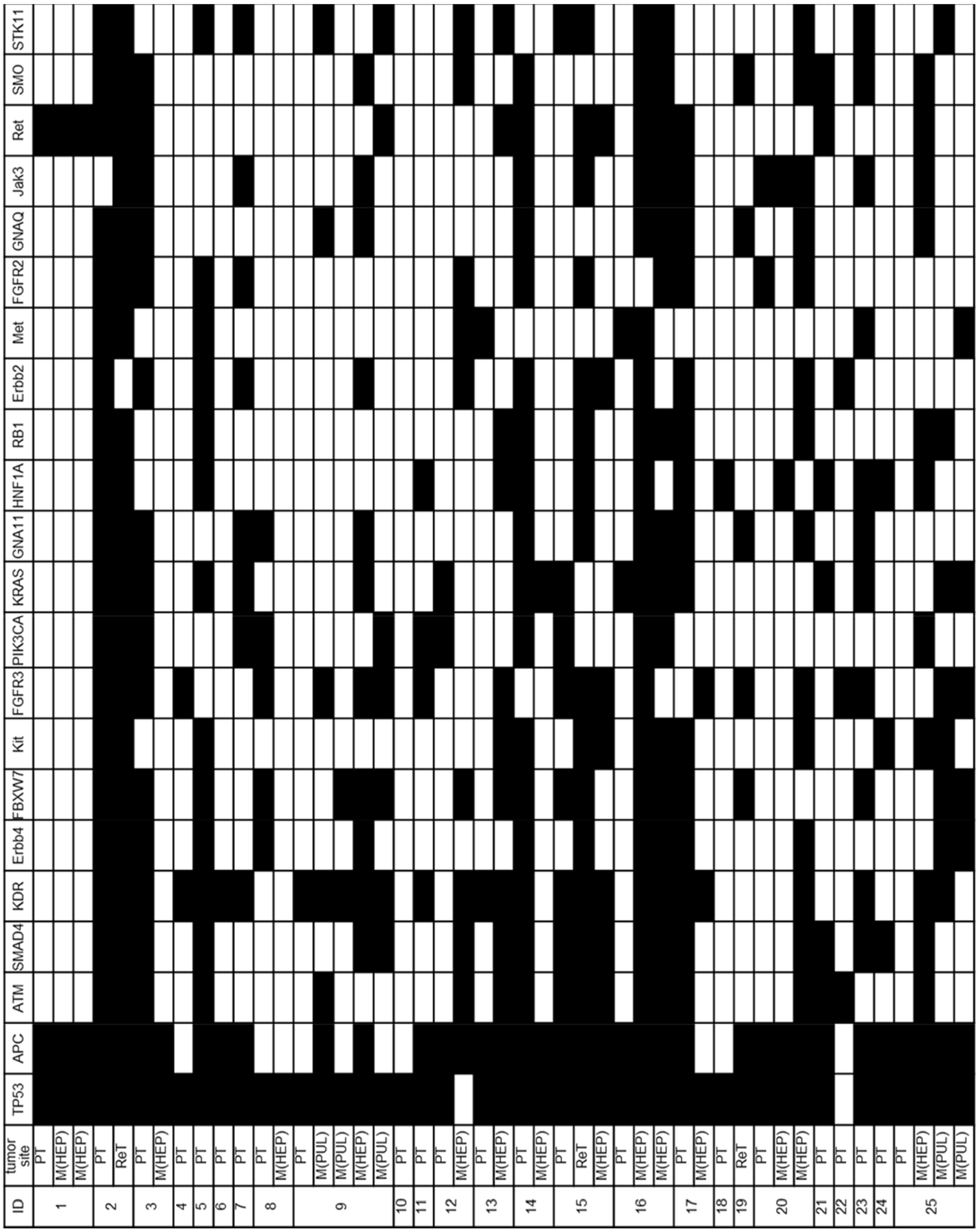

(*Continued*)

B

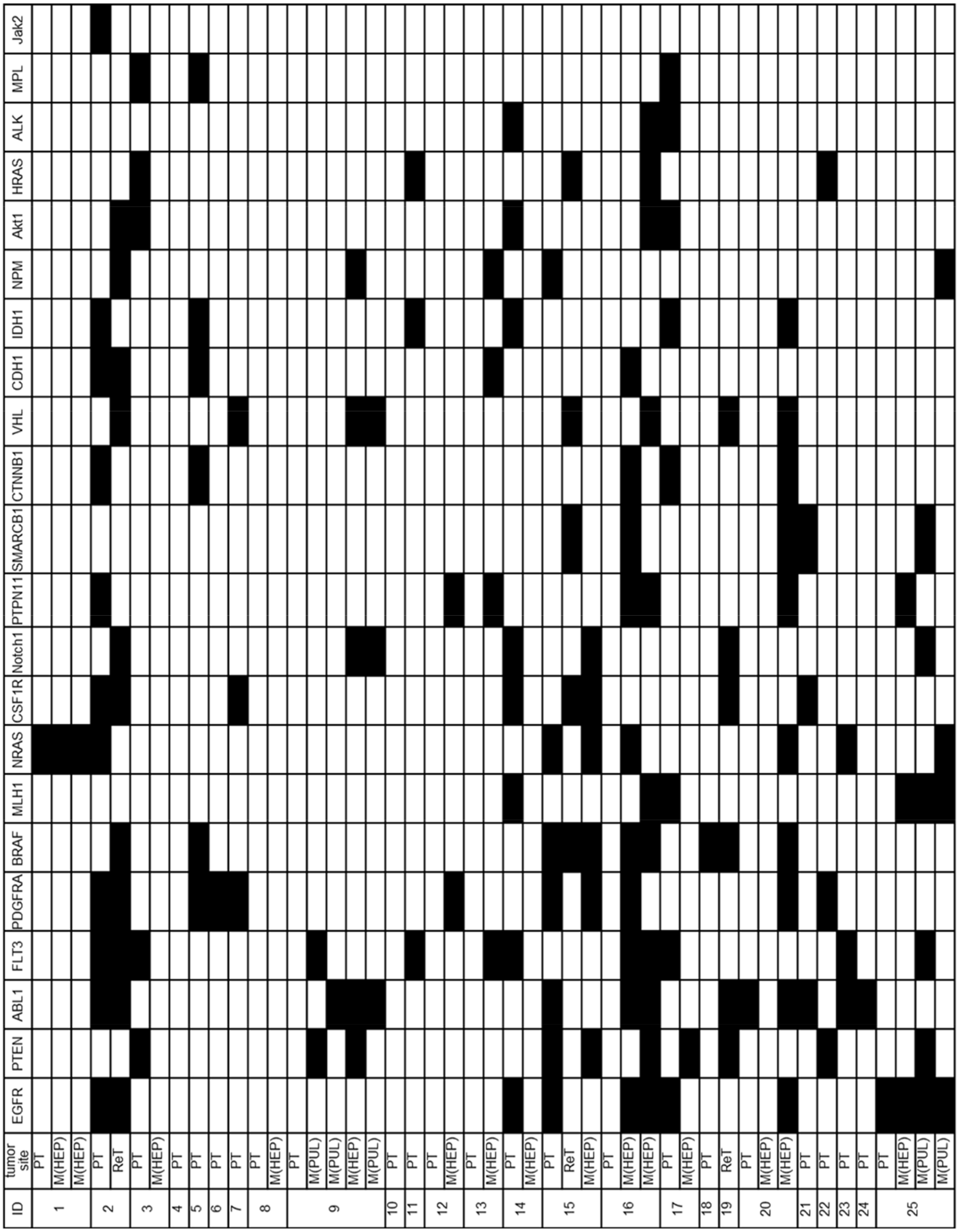
ID
tumor site
Jak2
MPL
ALK
HRAS
Akt1
NPM
IDH1
CDH1
VHL
CTNNB1
SMARCB1
PTPN11
Notch1
CSF1R
NRAS
MLH1
BRAF
PDGFRA
FLT3
ABL1
PTEN
EGFR
1 PT M(HEP) M(HEP)
2 PT ReT
3 PT M(HEP)
4 PT
5 PT
6 PT
7 PT
8 PT M(HEP)
9 PT M(PUL) M(PUL) M(HEP) M(PUL)
10 PT
11 PT
12 PT M(HEP)
13 PT M(HEP)
14 PT M(HEP)
15 PT ReT M(HEP)
16 PT M(HEP) M(HEP)
17 PT M(HEP)
18 PT
19 ReT
20 PT M(HEP) M(HEP)
21 PT
22 PT
23 PT
24 PT
25 PT M(HEP) M(PUL) M(PUL)

Supplementary Table 3: PIK3CA mutations detected in CRCs next to known "hotspot" codons 545, 549 and 1047

ID	PTU/M	exon	HGVSp	% AF	consequence	domain
3	PT	9	Ser541Phe	10.76	missense	helical
8	PT	9	Gln546Glu	11.0	missense	helical
12	PT	9	Glu545Lys	21.48	missense	helical
2	ReT	20	Gly1049Asp	10.09	missense	kinase
		20	Gly1050Ser	10.59	missense	kinase
14	PT	20	Asp1056Asn	13.93	missense	kinase
16	M(HEP)	20	Met1055Lys	14.21	missense	kinase
25	M(HEP)	20	Phe1059Leu	10.04	missense	kinase

The table shows non-"hotspot" PIK3CA mutations detected in primary/recurrent tumors or metastases. %AF= percent allele frequency.
